# Olfactory bulb-medial prefrontal cortex theta synchronization is associated with anxiety

**DOI:** 10.1038/s41598-024-63101-z

**Published:** 2024-05-27

**Authors:** Morteza Mooziri, Ali Samii Moghaddam, Mohammad Ali Mirshekar, Mohammad Reza Raoufy

**Affiliations:** 1 Student Research Committee, Zahedan University of Medical Sciences, Zahedan, Iran; 2 School of Medicine, Zahedan University of Medical Sciences, Zahedan, Iran; 3 Clinical Immunology Research Center, Zahedan University of Medical Sciences, Zahedan, Iran; 4 Department of Physiology, School of Medicine, Zahedan University of Medical Sciences, Zahedan, Iran; 5 Department of Physiology, Faculty of Medical Sciences, Tarbiat Modares University, Tehran, Iran; 6 Institute for Brain Sciences and Cognition, Faculty of Medical Sciences, Tarbiat Modares University, Tehran, Iran; 7 Present Address: Department of Brain and Cognitive Sciences, Cell Science Research Center, Royan Institute for Stem Cell Biology and Technology, ACECR, Tehran, Iran

**Keywords:** Olfactory bulb, Medial prefrontal cortex, Theta synchrony, Anxiety, Mechanism, Cognitive neuroscience, Emotion, Neural circuits

## Abstract

Anxiety is among the most fundamental mammalian behaviors. Despite the physiological and pathological importance, its underlying neural mechanisms remain poorly understood. Here, we recorded the activity of olfactory bulb (OB) and medial prefrontal cortex (mPFC) of rats, which are critical structures to brain’s emotional processing network, while exploring different anxiogenic environments. Our results show that presence in anxiogenic contexts increases the OB and mPFC regional theta activities. Also, these local activity changes are associated with enhanced OB-mPFC theta power- and phase-based functional connectivity as well as OB-to-mPFC information transfer. Interestingly, these effects are more prominent in the unsafe zones of the anxiogenic environments, compared to safer zones. This consistent trend of changes in diverse behavioral environments as well as local and long-range neural activity features suggest that the dynamics of OB-mPFC circuit theta oscillations might underlie different types of anxiety behaviors, with possible implications for anxiety disorders.

## Introduction

Anxiety is the emotional state in which an individual anticipates negative events, even without presence of an immediate threat^1–4^. From an evolutionary point of view, anxiety is believed to provoke behavioral responses that help the animal avoid the danger to ensure self-preservation^3^. However, the excess or generalization of these mental experiences can lead to pathological conditions^5^. This includes a range of phenomena such as psychiatric disorders in humans and specific behavioral patterns in animals^1^. Therefore, knowledge about such fundamental behavior has important implications both in health and disease. Unfortunately, the neural underpinnings of anxiety are still largely unknown.

There is a body of evidence describing the roles of medial prefrontal cortex (mPFC) as a major neural hub for fear and anxiety in mammals^1–3,5–13^. mPFC has long-range connections with several regions, such as basolateral amygdala (BLA) and ventral hippocampus (vHPC), forming the brain’s integrated network of anxiety processing^3,7,11^. In humans and non-human primates, the prefrontal cortex, that is expanded during evolution, actively modulates the level of anxiety^5^. Additionally, rodent studies show that anxiety is associated with augmentation of mPFC activity^2,8^. Specifically, presence in an anxiogenic environment is associated with mPFC theta activity enhancement^11^. Therefore, mPFC is a pivotal part of the anxiety processing network in the brain with local and long-range activities.

Previous studies show that olfactory bulb (OB) has important roles in cognitive and psychiatric phenomena^6,10,12,14–16^. OB, as a major modulator of emotional behaviors such as fear and anxiety, has anatomical connections to different brain structures, like mPFC and hippocampal formation, which are critical for cognitive and emotional processing^6,10,12,17,18^. In this line, it is shown that OB local activity is elevated in animals during fear^10,12^. Furthermore, human and animal studies show that allergic respiratory diseases, such as allergic rhinitis, are associated with cognitive and psychiatric abnormalities as well as disrupted brain activity^2,6,8,14,15,19–21^, which are suggested to be mediated by changes in OB function due to olfactory sensory neurons (OSNs) dysfunction^10,22–24^. Furthermore, OB activity is believed to be strongly entrained by respiration, a phenomenon which might have substantial contributions to different behaviors^10,12,18^. Thus, it seems like OB activity has important functions in modulating brain state and behavior.

Cognitive performance requires coherent activity of distinct brain regions, often referred to as the Communication-Through-Coherence (CTC) hypothesis^25,26^. In this line, neural oscillations have fundamental implications in synchronizing the activity between different brain structures^2,8,11,14,15,20^. Preceding works establish associations between enhanced theta synchronization in several brain circuits and cognitive performance or psychiatric status^2,8,11,15,18,20^. Specifically, the boosted theta activity in the anxiogenic conditions is accompanied by elevated theta connectivity between mPFC and major anxiety processing areas of the brain^2,7,11^. On the other hand, OB has functional connectivity with areas that process emotional and cognitive signals, an observation that is probably facilitated by respiration^6,10,12,14,15,18,27,28^. Also, it is shown that olfactory inputs actively modulate rodent mPFC activity and are required for fear expression^10,12,16^. Hence, inter-regional theta synchronization is among the essential neural codes of anxiety. While OB and mPFC are shown to have theta synchrony with other areas during aversive emotions^10,11^, their functional connectivity in the anxious state is yet to be explored.

Together, OB and mPFC are important nodes of the mammalian brain’s emotional processing network^2,3,6–8,10–13,16,29^. While mPFC modulations of anxiety behaviors are extensively studied, the roles of OB activity as well as the OB-mPFC circuit connectivity in anxiety-related processing are still matter of question. Here, we used a combination of behavioral and neurophysiological techniques to study the oscillatory features of the OB-mPFC circuit during different rodent anxiety behaviors.

Real-time simultaneous recording of OB and mPFC in distinct anxiogenic contexts revealed that presence in anxiogenic environments is associated with enhancement of OB and mPFC local theta (4–12 Hz) activities, as well as the OB-mPFC circuit theta synchrony. Interestingly, this theta synchrony is associated with increased information transfer from OB to mPFC. Furthermore, these hyperactivations are more pronounced in the unsafe states of the anxiogenic environments, compared to the safe states. Therefore, OB-mPFC circuit theta activities are among the fundamental neural correlates of anxiety behaviors in rodents.

## Results

### Neurophysiology and behavior

**Figure 1:**
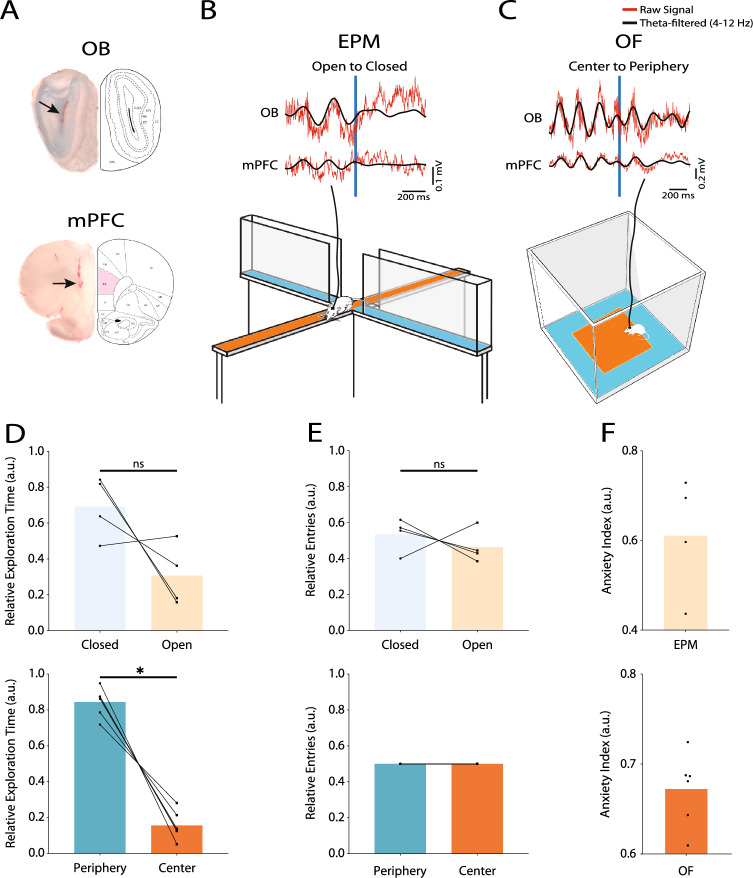
Recording sites and behavior. (**A**) Representative histological samples to confirm electrode sites at OB (upper panel) and mPFC (lower panel). Arrows point to the recording sites. (**B**, **C**) Schematic illustration of EPM (**B**) and OF (**C**), as the anxiogenic environments used in this study (lower panels). Sample of simultaneous real-time OB and mPFC raw (red traces) and theta-filtered (4–12 Hz) (black traces) LFPs while exploring each anxiogenic condition (upper panels). (**D**–**F**) Anxiety metrics reported as relative compartment exploration time (**D**), relative compartment entries (**E**), and anxiety index (**F**) in the EPM (upper panels) and OF (lower panels) experiments. Bars represent mean values. Statistical difference measured by Wilcoxon signed rank test. *p < 0.05. OB, olfactory bulb; mPFC, medial prefrontal cortex; LFP, local field potential; EPM, elevated plus maze; OF, open field.

We recorded simultaneous activity of OB and mPFC (Fig. 1A) as the animals explored two widely-studied mazes for evaluation of rodent anxiety, elevated plus maze (EPM; Fig. 1B) and open field (OF; Fig. 1C), following a baseline recording session. These apparatuses are shown to alter the activity of brain regions that process anxiety-related information, without affecting other areas^11^. Therefore, they are considered to specifically change brain circuits underlying anxiety-related behaviors. Furthermore, since locomotion can modulate theta activity of brain regions^11^, all the analyses of the present study were carried out on 5-s trials in which the animal had locomotor activity, for all three possible conditions, that is EPM, OF, and baseline. This approach, that is similarity in locomotor activity in all conditions, is previously reported to successfully detect changes in regional theta activities due to cognitive processing and cancel-out the changes related to locomotion^11^. Behaviorally, the animals spent a higher portion of their exploration time in safer compartments (EPM open vs. closed: 0.31 ± 0.09, 0.69 ± 0.09, respectively, p = 0.25, Cohen’s d = 1.12; OF center vs. periphery: 0.16 ± 0.03, 0.84 ± 0.03, respectively, p = 0.03, Cohen’s d = 4.29; Fig. 1D). However, we found no significant difference between safe or unsafe entries in neither experiments (EPM open vs. closed: 0.46 ± 0.05, 0.54 ± 0.05, respectively, p = 0.63, Cohen’s d = 0.38; since every transition in OF happens between different states, the proportion of entries to each compartment are the same; Fig. 1E). These results lead to the overall anxiety index (see “Methods”) of 0.61 ± 0.07 in the EPM experiment and 0.67 ± 0.02 in the OF experiment (Fig. 1F). Along with previous reports^3,30^, these observations show that EPM open arms and OF central area are more anxiogenic to rodents than closed arms and peripheral area, respectively.

### Anxiogenic environments enhance OB and mPFC *theta* activity

**Figure 2:**
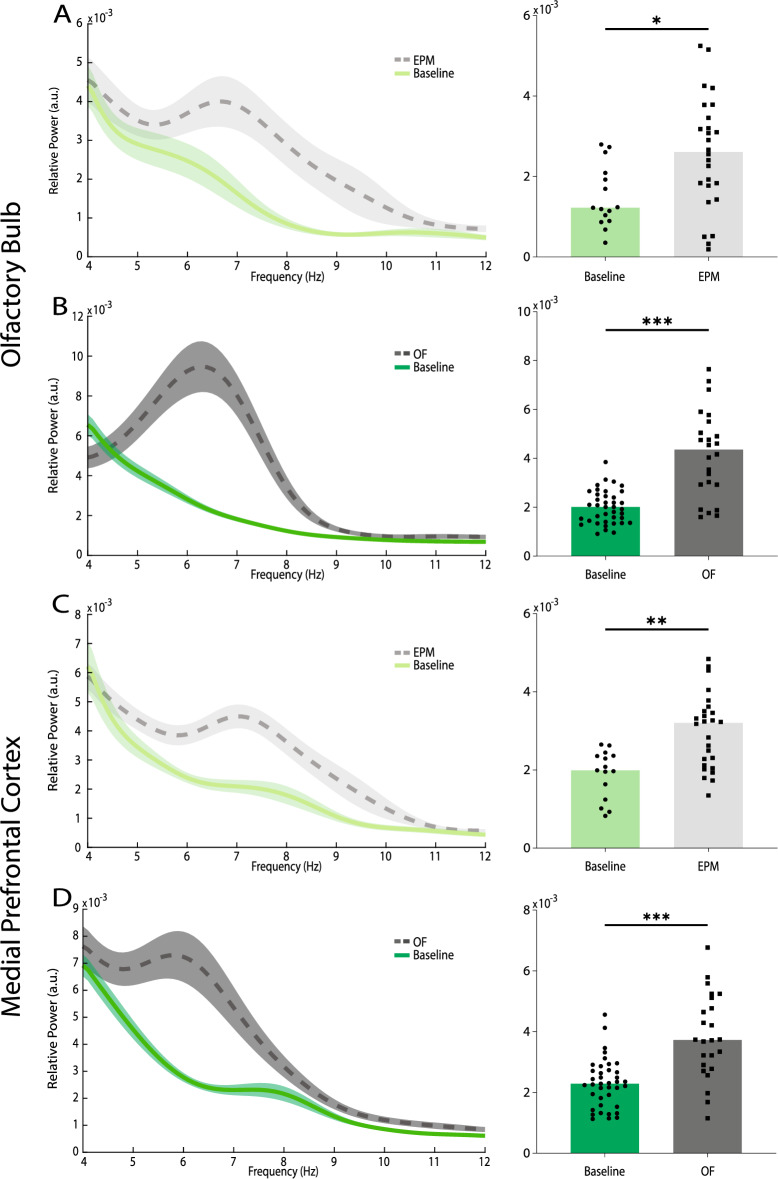
OB and mPFC theta power during anxiety. (**A**–**D**) Theta PSD (left panels) and average power (right panels) of OB (**A**, **B**) and mPFC (**C**, **D**) in the EPM (n = 26) (**A**, **C**) and OF (n = 24) (**B**, **D**) experiments (baseline: n = 15, n = 39, respectively). Data presented as mean ± SEM in the PSDs. Bars represent median values in the bar plots. Statistical difference measured by Mann–Whitney test. *p < 0.05, **p < 0.01, ***p < 0.001. PSD, power spectral density; OB, olfactory bulb; mPFC, medial prefrontal cortex; EPM, elevated plus maze; OF, open field.

As previously shown, local theta activity of brain regions that process anxiety increases in anxiogenic environments^11^. To explore whether anxiogenic contexts can enhance the OB and mPFC local theta oscillations, we compared the activity of these two regions in each context, regardless of the zone, with the familiar condition for the animal, that is home cage. The results of power spectral density (PSD) analysis show that both behavioral conditions significantly elevated the theta power of OB (EPM vs. baseline: 2.57 × 10^–3^ ± 2.74 × 10^–4^, 1.50 × 10^–3^ ± 1.99 × 10^–4^, respectively, p = 0.009; OF vs. baseline: 4.17 × 10^–3^ ± 3.62 × 10^–4^, 2.00 × 10^–3^ ± 1.10 × 10^–4^, respectively, p < 0.001; Fig. 2A, B) and mPFC (EPM vs. baseline: 2.98 × 10^–3^ ± 1.86 × 10^–4^, 1.89 × 10^–3^ ± 1.60 × 10^–4^, respectively, p = 0.001; OF vs. baseline: 3.84 × 10^–3^ ± 2.83 × 10^–4^, 2.30 × 10^–3^ ± 1.28 × 10^–4^, respectively, p < 0.001; Fig. 2C, D). These findings are consistent with previous reports on mPFC theta activity in anxiogenic environments^11^, as well as OB and mPFC activity during an aversive emotional state, particularly conditioned fear^7,10,13^.

Beyond that, hippocampal theta sub-bands are specialized for distinct purposes, with slower oscillations for emotional and faster ones for cognitive processing^31,32^. Human and rodent studies also support this notion for emotional processing in other brain areas, like BLA, mPFC, and OB^10,12,13^. Therefore, we sought to find possible differences between low (4–8 Hz) and high (8–12 Hz) theta activities in OB and mPFC during anxiety. Interestingly, we observed that OB (EPM: low theta: Cohen’s d = 0.76, p = 0.02, high theta: Cohen’s d = 0.65, p = 0.01; OF: low theta: Cohen’s d = 1.79, p < 0.001, high theta: Cohen’s d = 0.97, p < 0.001; Fig. 2A, B and Supplementary Fig. 1A, B) and mPFC (EPM: low theta: Cohen’s d = 1.19, p = 0.002, high theta: Cohen’s d = 0.70, p = 0.01; OF: low theta: Cohen’s d = 1.45, p < 0.001, high theta: Cohen’s d = 0.72, p = 0.02; Fig. 2C, D and Supplementary Fig. 1C, D) low theta enhancements were more robust than high theta, in both behavioral paradigms. Collectively, OB and mPFC theta oscillations might be heightened during anxiogenic states, even without presence of the evident threat. Moreover, these changes are more prominent in the slow theta oscillations, compared to fast theta.

### Anxiogenic environments synchronize OB-mPFC circuit *theta* oscillations

**Figure 3:**
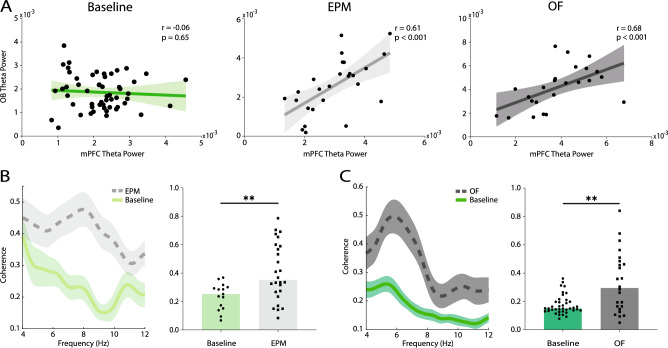
OB-mPFC circuit theta synchrony during anxiety. (**A**) OB-mPFC theta power correlation in baseline (leftmost panel, n = 54), EPM (middle panel, n = 26), and OF (rightmost panel, n = 24). Correlation analyzed by spearman’s rank method (shaded area identifies 95% CI). (**B**, **C**) OB-mPFC theta spectral coherence (left panels) and average theta coherence (right panels) in EPM (n = 26) (**B**) and OF (n = 24) (**C**) (baseline: n = 15, n = 39, respectively). Data presented as mean ± SEM in the spectral coherence. Bars represent median values in the bar plots. Statistical difference measured by Mann–Whitney test. **p < 0.01. OB, olfactory bulb; mPFC, medial prefrontal cortex; EPM, elevated plus maze; OF, open field; CI, confidence interval.

Theta band oscillations are among the most essential means which the brain uses for inter-regional synchronization^18,33–37^. In the anxiogenic environments, theta connectivity happens between brain structures that are critical for the expression of anxiety behaviors, with other circuits remaining untouched^11^. Here, we used power correlation, which is a measure of inter-regional power-based connectivity, to assess the OB-mPFC circuit theta synchrony for the three conditions. The results show that OB and mPFC theta power are not correlated in the baseline condition (r = − 0.06, p = 0.65, Spearman correlation; Fig. 3A). However, once the animals are placed in the anxiogenic contexts, a significant theta power correlation appears in the OB-mPFC circuit (EPM: r = 0.61, p < 0.001; OF: r = 0.68, p < 0.001; Spearman correlation; Fig. 3A). Using Fisher r-to-z transformation, we found that the theta power correlation in the EPM and OF were significantly greater than the baseline condition (p = 0.002, p < 0.001, respectively).

To further explore this issue, we computed theta coherence between these regions, which is a phase-based connectivity method and is reported to be a neural code of psychiatric phenomena^2,8,10,11,20^. OB-mPFC theta coherence was significantly higher in the EPM (maze = 0.41 ± 0.04, baseline = 0.24 ± 0.02, p = 0.006; Fig. 3B) and OF (maze = 0.33 ± 0.04, baseline = 0.17 ± 0.01, p = 0.004; Fig. 3C) compared to familiar condition. To exclude the effect of power enhancement on theta phase coherence, we also computed OB-mPFC theta phase-locking value (PLV; see “Methods”), which led to results similar to coherence (EPM vs. baseline: 0.50 ± 0.03, 0.34 ± 0.02, respectively, p = 0.001; OF vs. baseline: 0.44 ± 0.04, 0.28 ± 0.01, respectively, p = 0.001; Supplementary Fig. 2). This indicates that coherence results are not biased due to changes in power. Consistent with previous reports^6,10^, and the results of regional neural activity presented above, these observations suggest that presence in anxiogenic conditions enhances the OB-mPFC circuit theta synchrony.

### OB-mPFC circuit *theta* oscillations during anxiety are not due to locomotion

Theta oscillations are susceptible to being driven by locomotion, as a behavioral state^11^. To evaluate the roles of locomotion on the OB-mPFC circuit theta enhancements during EPM and OF exploration, we searched for the relationship between the animals’ movement and the OB-mPFC circuit theta features. We observed that the animals’ speed during EPM, but not OF, exploration was higher than baseline (EPM vs. baseline: 7.31 ± 0.32, 5.78 ± 0.52, respectively, p = 0.02; OF vs. baseline: 6.31 ± 0.47, 6.20 ± 0.33, respectively, p = 0.81; Supplementary Fig. 3).Next, we hypothesized that if locomotion is driving a region’s activity or the circuit connectivity, there should be a positive correlation between that feature and the animal’s speed. We found that there is no significant correlation between the animal’s speed and OB (Baseline: r = − 0.16, p = 0.25; EPM: r = − 0.28, p = 0.17; OF: r = 0.14, p = 0.53; Spearman correlation; Supplementary Fig. 4A–C) or mPFC (Baseline: r = 0.16, p = 0.25; EPM: r = − 0.36, p = 0.07; OF: r = 0.13, p = 0.56; Spearman correlation; Supplementary Fig. 4A–C) theta power in none of the experimental conditions, namely baseline, EPM and OF. We also observed that the OB-mPFC theta coherence is not correlated with movement (Baseline: r = − 0.11, p = 0.43; EPM: r = − 0.06, p = 0.76; OF: r = 0.26, p = 0.23; Spearman correlation; Supplementary Fig. 4A–C). Therefore, theta enhancements in the OB-mPFC circuit while exploring EPM and OF are not explained by the animal’s locomotion. Considering previous literature^11^, these results suggest that the above-described OB-mPFC circuit activations are most probably due to the animal’s experience of anxiety, rather than the effects of locomotion, as a behavioral state.

### OB leads mPFC during anxiety

**Figure 4:**
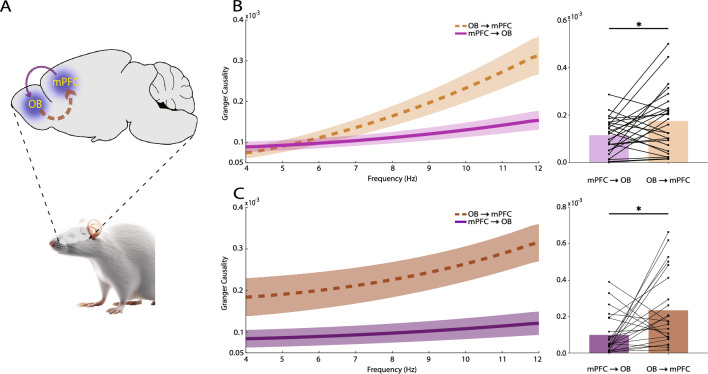
Theta information transfer between OB and mPFC during anxiety. (**A**) Schematic sagittal section to illustrate OB and mPFC locations and the investigated directionalities. (**B**, **C**) OB-to-mPFC and mPFC-to-OB theta spectral (left panels) and average (right panels) GC in EPM (n = 26) (**B**) and OF (n = 24) (**C**). Data presented as mean ± SEM in the spectral GC. Bars represent mean values in the bar plots. Statistical difference measured by Wilcoxon signed rank test. *p < 0.05. OB, olfactory bulb; mPFC, medial prefrontal cortex; GC, Granger causality; EPM, elevated plus maze; OF, open field.

Mechanistically, OB inputs actively modulate prefrontal activity during episodes of fear^10,12,16^. It is shown that during conditioned fear, OB-to-mPFC information transfer is significantly higher than the inverse direction, that is mPFC-to-OB^12^. Hence, we tried to investigate the theta band information transfer between OB and mPFC during anxiety (Fig. 4A), using Granger causality (GC), which measures the predictability of one time-series based on another^38,39^. To explore these effects, firstly, it is required to make sure whether there is GC effect in each of the mentioned directions, which was done through manual permutation (see “Methods”). The results of the permutation analyses confirm that there is bidirectional theta information transfer between OB and mPFC in EPM (OB-to-mPFC: permutation distribution = 4.28 × 10^–5^ ± 2.24 × 10^–6^, data mean = 2.91 × 10^–4^, z-score = 4.94; mPFC-to-OB: permutation distribution = 2.47 × 10^–5^ ± 1.06 × 10^–6^, data mean = 1.43 × 10^–4^, z-score = 4.98; Supplementary Fig. 5A, B). This effect was seen only in the OB-to-mPFC direction in OF (OB-to-mPFC: permutation distribution = 6.75 × 10^–5^ ± 3.75 × 10^–6^, data mean = 5.25 × 10^–4^, z-score = 5.47; mPFC-to-OB: permutation distribution = 3.91 × 10^–5^ ± 1.51 × 10^–6^, data mean = 9.66 × 10^–5^, z-score = 1.70; Supplementary Fig. 5C, D). Subsequently, we sought to figure out whether there is stronger information flow in any of these directions during anxiety. The results show that there is significantly greater theta signal propagation in the OB-to-mPFC direction, compared to mPFC-to-OB, in EPM (OB-to-mPFC = 1.75 × 10^–4^ ± 2.54 × 10^–5^, mPFC-to-OB = 1.15 × 10^–4^ ± 1.45 × 10^–5^, p = 0.04; Fig. 4B) and OF (OB-to-mPFC = 2.35 × 10^–4^ ± 4.18 × 10^–5^, mPFC-to-OB = 9.92 × 10^–5^ ± 3.32 × 10^–5^, p = 0.03; Fig. 4C). Along with previous reports on OB modulatory effects on mPFC^10,12,16^ and OB-to-mPFC information flow^12^ during freezing behavior, our data suggest that OB theta oscillations convey key signals to mPFC, facilitating the expression of anxiety behaviors.

### OB-mPFC circuit *theta* enhancements are more prominent in the unsafe zones of the anxiogenic environments

**Figure 5:**
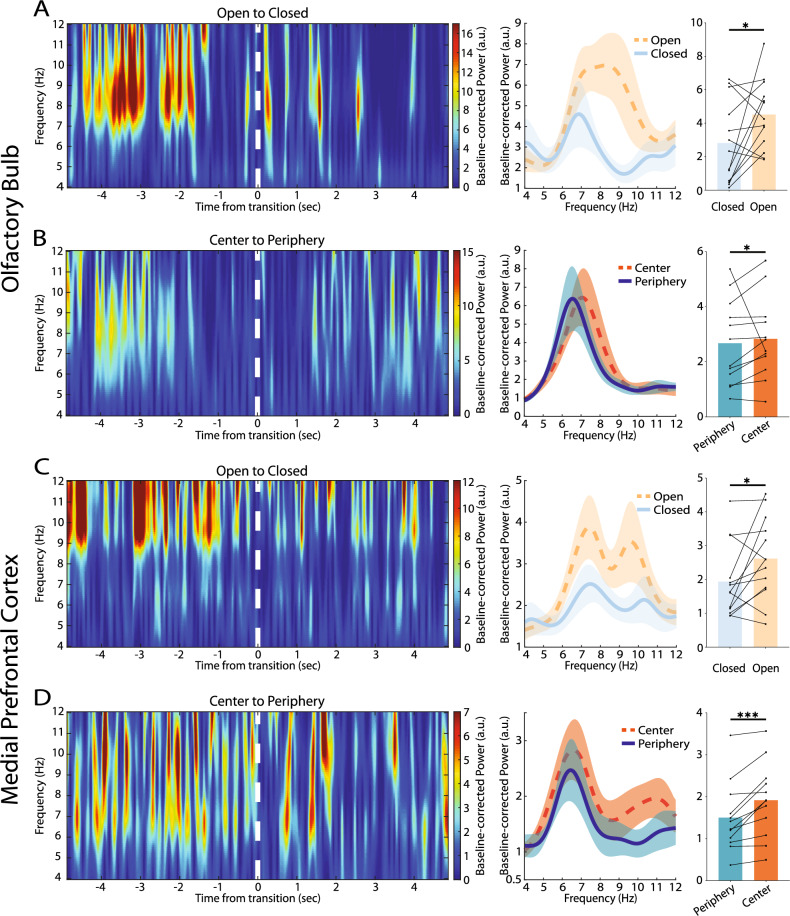
OB and mPFC theta power in different states of the anxiogenic environments. (**A**–**D**) Representative spectrogram (leftmost panels), PSD (middle panels), and average power (rightmost panels) of OB (**A**, **B**) and mPFC (**C**, **D**) theta activity in the EPM (n = 13) (**A**, **C**) and OF (n = 12) (**B**, **D**). Warmer colors in the spectrograms indicate greater values of baseline-corrected power. Data presented as mean ± SEM in the PSDs. Bars represent mean values in the bar plots. Statistical difference measured by Wilcoxon signed rank test. *p < 0.05, ***p < 0.001. PSD, power spectral density; OB, olfactory bulb; mPFC, medial prefrontal cortex; EPM, elevated plus maze; OF, open field.

Distinct areas of EPM and OF induce different levels of anxiety in rodents; EPM open arms and OF central area are more anxiogenic compared to closed arms and periphery, respectively^3,30^. Thus, we searched for the neural correlates of these behavioral phenomena in the OB-mPFC circuit. The results show that OB theta power enhancement is significantly greater for the unsafe zones compared to safer zones, both in the EPM (open = 4.52 ± 0.57, closed = 2.81 ± 0.68, p = 0.04; Fig. 5A) and the OF (center = 2.83 ± 0.43, periphery = 2.67 ± 0.45, p = 0.03; Fig. 5B). A similar trend of changes were observed for mPFC (EPM: open = 2.62 ± 0.34, closed = 1.94 ± 0.30, p = 0.03; OF: center = 1.92 ± 0.26, periphery = 1.50 ± 0.24, p < 0.001; Fig. 5C, D). We also observed that these effects were present in the safe-to-unsafe transitions in OF (OB in center vs. periphery: 2.94 ± 0.45, 2.27 ± 0.50, respectively, p = 0.046; mPFC in center vs. periphery: 1.98 ± 0.30, 1.28 ± 0.16, respectively, p < 0.001; Supplementary Fig. 6), while they did not reach significance in EPM in this setting (OB in open vs. closed: 4.70 ± 1.36, 3.90 ± 0.67, respectively, p = 0.32; mPFC in open vs. closed: 2.57 ± 0.82, 2.58 ± 0.50, respectively, p = 0.37; Supplementary Fig. 6). Therefore, beyond the results presented above, these observations propose that OB and mPFC regional theta activity enhancements in the anxiogenic environments are also sensitive to presence of the evident threat.

**Figure 6:**
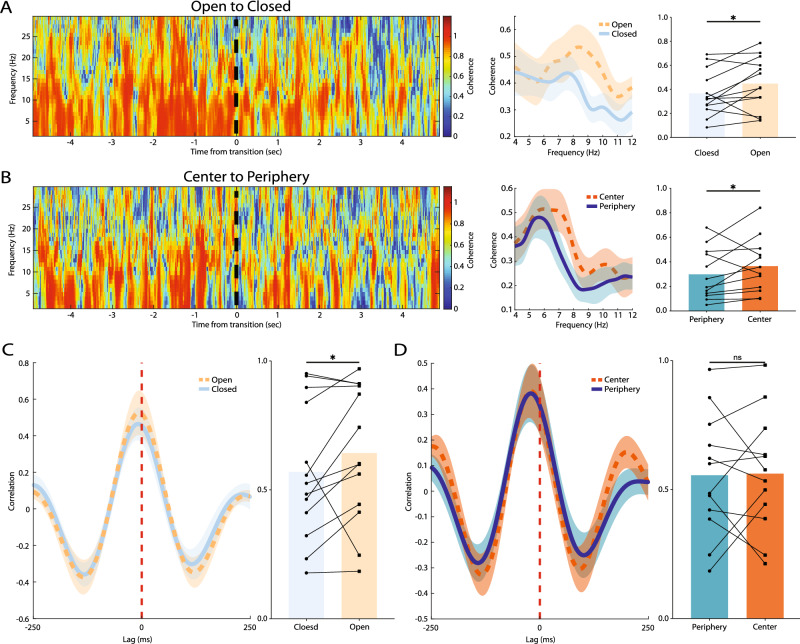
OB-mPFC circuit theta synchrony in different states of the anxiogenic environments. (**A**, **B**) OB-mPFC theta coherogram (leftmost panels), spectral (middle panels), and average (rightmost panels) coherence in EPM (n = 13) (**A**) and OF (n = 12) (**B**). (**C**, **D**) OB-mPFC theta cross-correlation (left panels) and peak values (right panels) in EPM (**C**) and OF (**D**). Warmer colors in the coherograms indicate greater values of coherence. Data presented as mean ± SEM in the spectral coherence and cross-correlation plots. Bars represent mean values in the bar plots. Statistical difference measured by Wilcoxon signed rank test. *p < 0.05. OB, olfactory bulb; mPFC, medial prefrontal cortex; EPM, elevated plus maze; OF, open field; CI, confidence interval.

To further discern the neural correlates of unsafe and safe zones of the anxiogenic environments, we explored the inter-regional connectivity within the OB-mPFC circuit during different states of each behavioral paradigm. Interestingly, theta coherence was significantly higher in the unsafe zones, compared to safer zones in the two behavioral paradigms (EPM: open = 0.45 ± 0.06, closed = 0.37 ± 0.05, p = 0.03; OF: center = 0.37 ± 0.06, periphery = 0.30 ± 0.06, p = 0.046; Fig. 6A, B). Also, OB-mPFC cross-correlation was significantly enhanced in the open arms of EPM compared closed arms (open = 0.64 ± 0.07, closed = 0.57 ± 0.07, p = 0.04; Fig. 6C). However, we did not observe this effect in the OF (center = 0.56 ± 0.07, periphery = 0.56 ± 0.07, p = 0.43; Fig. 6D). Consistent with previous results, these functional connectivity enhancements further emphasize the impact of OB-mPFC theta oscillations in anxiety-related information processing.

To figure out whether or not the power and coherence difference between safe and unsafe states of each anxiogenic environment are due to changes in locomotor activity, we also compared the animals’ speed during transitions. Hypothetically, higher speed in the unsafe states, compared to safer states could potentially propose that these between-state changes could be due to locomotion. We observed that the animals’ speed was significantly lower in the open arms of EPM, compared to the closed arms (open = 6.48 ± 0.53, closed = 8.16 ± 0.20, p = 0.02; Supplementary Fig. 7A). In the OF, we found that there is no significant difference between animals’ speed in the center and periphery of the maze (center = 6.04 ± 0.63, periphery = 6.58 ± 0.72, p = 0.52; Supplementary Fig. 7B). These observations suggest that the more prominent OB-mPFC theta oscillations in the unsafe states of the anxiogenic environments are likely due to emotional processing within this circuit, rather than animal’s locomotion.

## Discussion

Here we show that presence in anxiogenic contexts enhances theta oscillations of the OB-mPFC circuit. Anxiety behaviors are associated with enhancement of OB and mPFC regional theta activity. Furthermore, these local activity enhancements are associated with increased inter-regional connectivity in the same frequency band. We also observed that OB delivers key information to mPFC during episodes of anxiety. Interestingly, these local and long-range activity patterns are more pronounced in the unsafe zones of the two behavioral paradigms. These findings, along with previous reports on the roles of this circuit in processing aversive emotions, propose that OB-mPFC circuit theta activities are among the important neural correlates of anxiety within the rodent brain.

mPFC is found to be a critical structure in modulating the expression of emotional behaviors^3,16^. Rodent^7,10,12^ and human^13^ studies show that processing fearful cues requires mPFC activity as well as its precise long-range communications with other brain areas. In this line, mPFC is thought to have coordinated activity with structures like amygdala and vHPC, a collaboration which primarily aims at determining the presence of threatening conditions^16^. Specifically, it is demonstrated that presence in anxiogenic contexts increases mPFC activity^11^. Moreover, enhancement of resting-state mPFC activity is accompanied by pathological anxiety levels in animals^2,6,8,20^. Importantly, many of these activities are reported to happen in the theta frequency band or sub-bands^2,6,8,10–13^. Well-aligned with previous reports, our data show that mPFC theta activity increases in the anxiogenic environments. Furthermore, this enhancement is sensitive to the state of the environments, with higher activations in the unsafe zones, where the threat is actual, compared to the safer zones, where there is an abstract threat. After detection of a danger, subsequent pathways, areas, and circuits are employed to finely express the most appropriate emotional response^16^. Therefore, this would be interesting for future studies to see how the activity of these downstream areas are formed in response to unsafety to form the animal’s reaction. Together, our data further support the idea that mPFC theta activity is a canonical neural rhythm of anxiety, probably underlying a broad range of anxiety-related behaviors and disorders.

There is a body of evidence demonstrating the roles OB in cognitive and psychiatric processing^6,10,12,14,15,18,27^. Specifically, preceding works show that OB theta activities are enhanced during learned fear in rodents^10,12^. On the other hand, animal models of allergic respiratory diseases, for instance allergic rhinitis, also show higher level of anxiety^2,6,8,20^, which is associated with enhancement of resting-state OB activity^6^. However, despite this evidence, we are lacking investigations on real-time activity of OB during anxiety. Importantly, this approach will provide direct experimental evidence to further advance our current understanding of the anxiety processing mechanisms. Here, we show that OB theta oscillations are enhanced in distinct types of anxiety. Moreover, this enhancement is larger as the unsafety increases. Mechanistically, it is shown that OB roles in fear expression are dependent on nasal respiration, most-probably due to mechanical stimulation of OSNs in the nasal mucosa^10,12,24^. Specifically, respiration-OB theta connectivity, which is probably a major driver of OB oscillations, is elevated in a wide range of behaviors^12,18^. Thus, in case of anxiety, it would also be of interest to see if nasal respiration has modulatory effects on the behavior and the activity of downstream brain regions receiving OB inputs. Together, along with previous reports^10,12^, we suggest that OB is a significant contributor to the brain’s emotional processing mechanisms. However, further studies are required to investigate its roles in processing emotions, specifically anxiety.

mPFC is a higher cognitive area within the mammalian brain; thus, it is expected that every prefrontal rhythm contributes to different behaviors. In this view, the precise tuning of the rhythm might provide a fine dissection between diverse behaviors. Previous evidence show that emotional and cognitive processes recruit distinct theta sub-bands of the vHPC, with the slower theta oscillations receiving the major credit for expression of emotional behaviors^31,32^. This idea is further supported by animal^7,10,12^ and human^13^ studies in which lower theta frequencies are shown to underlie fear. Importantly, these activities are reported in several brain regions, including vHPC, BLA, mPFC, and OB^7,10,12,13^, all of which are critical structures for the emotional processing network. Thus, it might be expected to see this activity pattern in the mPFC during anxiety. In fact, we observed that this notion is the case not only for mPFC, but also for the OB theta activity. Specifically, our data show that the enhancement of low theta activity is larger than high theta during episodes of anxiety, for both mPFC, as expected, and OB, surprisingly (see Supplementary Fig. 1). These effects were consistent throughout both behavioral paradigms. Complementary to previous reports^7,10,12,13^, our data suggest that OB and mPFC low theta neural activities might play a more prominent role in the expression of emotions, particularly anxiety here, compared to high theta. Therefore, we propose that slow theta oscillations might be a ubiquitous neural rhythm of aversive emotions within the rodent brain, and probably evolutionary higher mammals, arising from widespread brain areas. Additionally, we observed less strong high theta power enhancements, compared to low theta, in OB and mPFC, which was more clearly evident in EPM (see Fig. 5), suggesting that high theta enhancements are also associated with anxiety, however probably weaker. Minor enhancement of faster theta activities suggests that these oscillations might have minor, yet remarkably important, contributions to emotional processing. For instance, these rhythms might have a role for finely-tuned discrimination of behaviors. Further studies are required to dissect the roles of low and high theta neural activities of different brain areas in cognitive and emotional processes.

Many, and probably all, behaviors require synchronized activity of several brain regions^3,7,11–14,16,18,21,25,28,34^. In this line, theta oscillations have a major role for precisely tuning and coordinating the activity of neighboring or distant areas in a variety of cognitive and psychiatric phenomena^7,9,11–13,18^. Both mPFC and OB are found to have substantial functional connectivity with several brain regions^2,6–8,10–15,18,21,28^. Notably, these synchronizations are shown to encode diverse behaviors^6,7,10–15,18,21,28^. Our results show that OB-mPFC theta connectivity increases during anxiety. It is previously shown that presence in these anxiogenic environments enhances the functional connectivity between areas that process anxiety-related information, such as vHPC and mPFC^11^. Interestingly, areas that are probably less related to emotions, like dorsal HPC, remain spared in these conditions^11^. Furthermore, OB has direct and/or indirect connections to widespread brain structures, including but not limited to, amygdala, entorhinal cortex, hippocampus, and mPFC^18,27^. These and other areas receiving input from OB have significant roles in a vast variety of brain functions, from sensation to cognition^6,10,12,14,15,18,27,37,40^. It is even demonstrated that OB inputs are critical to brain’s circuit development^28^; importantly, absence of these inputs will cause deficits in structure, function, and behavior^28^. In line with these reports, we observed that OB transmits key information to mPFC in the theta frequency band during anxiety. This finding makes more sense when considered with previous evidence which present the OB modulations over prefrontal activity during learned fear^10,12^. Therefore, these results suggest that OB might have key roles in modulating the activity of downstream regions to form diverse brain functions and behaviors.

It is known that expression of each emotional behavior requires concerted pattern of activities from different brain structures^3,16,18^. Also, each area and circuit has varied contributions to different emotions^16^. This rich neural coding repository will lead to fine discrimination of specific behaviors in every category of behaviors. This view will provide a deeper insight for understanding the neural circuits underlying diverse anxiety-related phenomena. Particularly, it is plausible to think that there might be circuits and structures which code the category, here anxiety, and some others to underlie specific behaviors, for example acrophobia, rather than the whole category; we’d call them “domain-general circuits” and “behavior-specific circuits”, respectively. Contrarily to this discrete classification, these circuits might act in a continues domain. In this framework, all circuits participate in the expression of all behaviors; however, the extent to which they contribute to each behavior might differ. With this notion, theoretically, all the intuitions that are appliable to the microscale high-dimensional neural space might be also appliable to the macroscale global brain state. Here, we show that dynamics of OB-mPFC circuit theta oscillations are well-preserved in two different behavioral assays for rodent anxiety. Since these two paradigms are designed based on distinct features of rodents^30^, they might capture diverse types of anxiety. We can interpret these findings from two main aspects. First, from the physiological and basic science view, it might suggest that the two most-commonly used behavioral paradigms for rodent anxiety are comparable to one another, meaning that they have roughly equal sensitivity to detect the animal’s level of anxiety. Second, from the pathological and clinical view, these results might point to the OB-mPFC circuit theta enhancement as a general neural code of anxiety, appliable to, or maybe underlies, a variety of anxiety behaviors, as well as disorders such as generalized anxiety disorder, social anxiety disorder, or specific phobias. We are not able to confirm these theoretical interpretations in the current study settings, and thus strongly encourage further investigations to pursuit these ideas.

This study faces some limitations which need to be addressed. First, our data does not provide causal effects between anxiety and OB-mPFC circuit theta activities. Future works using perturbation methods, such as chemo- or opto-genetics, might help finding the causal influences between anxiety and OB-mPFC circuit theta oscillations. Second, we did not record the spiking activity of OB and mPFC neurons. It is important to see how these changes in the field activities will affect the neuronal functions of these regions. It is previously shown that theta activity in LFP is correlated with neuronal population activity, and not single neuron activity, for different tasks^41^. For instance, it would be of interest to figure out if the OB-to-mPFC theta information transfer affects the prefrontal neuronal function. Third, we did not study the roles of respiration and OSNs in this process. The respiration-to-OB signals mediated by OSNs are shown to have important roles in the expression of emotions^10,12^. Therefore, studies on the roles of respiration and OSNs could potentially provide interesting insights.

In sum, this study lays foundations for the roles of OB-mPFC communication in anxiety behaviors, and possibly anxiety disorders. We suggest that enhancement of OB and mPFC theta oscillations are associated with the expression of anxiety behaviors in rodents. Also, we show that theta frequency band plays a key role in synchronizing this circuit during anxiety by amplifying OB-mPFC functional connectivity as well as OB-to-mPFC information transfer. Along with previous reports, these data propose two major hypotheses. First, the OB-mPFC circuit might be an important contributor to the brain’s integrated network of anxiety processing, with possible implications for other aversive emotions. Second, OB might have critical modulatory effects on different brain areas for emotional or cognitive processing which might happen through direct and/or indirect anatomical and/or functional communications. Future investigations are required to pursuit these leads.

## Methods

### Animals

Twelve pathogen-free male Wistar rats (8–10 weeks of age, weighing 200–250 g) were taken from Laboratory Animals Research Center at Zahedan University of Medical Sciences (Zahedan, Iran). Standard laboratory conditions as 21 ± 2 °C temperature, 12:12 h light/dark cycle, and ad libitum access to food and water were prepared. All procedures were executed according to the ARRIVE guidelines 2.0^42^ and the National Research Council's Guide for the Care and Use of Laboratory Animals, and were confirmed by the “Ethics Committee of Zahedan University of Medical Sciences” (IR.ZAUMS.REC.1399.443).

### Surgery

Animals were anesthetized by a combination of ketamine (100 mg/kg) and xylazine (10 mg/kg), injected intraperitoneally. At the beginning of the surgery, an external heating pad was placed beneath the animal’s body to preserve temperature at 37 °C. Depth of anesthesia was checked by tail and pinch reflexes. Subsequently, the animal was fixed in a stereotaxic apparatus (Narishige, Japan). Local anesthesia of scalp region was provided by subcutaneous injection of lidocaine chlorhydrate 2% (0.5 ml). We then inserted stainless-steel recording electrodes (127 μm in diameter, A.M. system Inc., USA) into OB (AP: 8.5 mm, ML: − 1 mm, DV: − 1.5 mm) and Prelimbic mPFC (AP: + 3.2 mm; ML: − 0.6 mm; DV: − 3.6 mm) of the left hemisphere. A stainless-steel screw was implanted at the right parietal bone as the reference. Electrodes were joined in a socket that was fixed on the animal’s head using dental cement. Surgery’s local skin was disinfected by tetracycline. Surgical procedures were completed by injection of buprenorphine (0.1 mg/kg) as analgesic and sterile saline (1.0 ml) for hydration. Then, the animals were carried to home cage for recovery.

### Electrophysiology

A week after the surgery, real-time LFPs were recorded from OB and mPFC during behavioral tests. For this, animal’s head-socket was attached to a miniature buffer head stage with high-input impedance (BIODAC-A, TRITA Health Tec. Co., Tehran, Iran), through cables to a main AC coupled amplifier (1000 amplification) and the recording system (BIODAC-Bi401l9B, TRITA Health Tec. Co., Tehran, Iran). Simultaneous LFPs were recorded from OB and mPFC (low-pass filtered < 250 Hz, digitized at 2 kHz) and were analyzed offline using MATLAB software (The Mathworks Inc., USA).

### Behavior

The animals were habituated to the recording chamber one week before and after the surgery. Two recording sessions were conducted for each animal at 7–8 days after surgery (both on the same day). The first recording session was planned to capture each animal’s OB and mPFC baseline activity. Therefore, we recorded simultaneous LFPs from OB and mPFC while the animal freely explored a familiar arena, that is home cage (40 × 30 cm, 25 cm-high wall), for 10 min. The second recording session was designed to study anxiety state using either EPM or OF. So, the animals were randomly divided into two groups (n = 6 per group). The first group were placed on the center of the EPM (50 cm height from the floor, 50 cm-high wall, with four 50 × 10 cm arms perpendicular to each other; Fig. 1B), and were allowed to freely explore the environment for 10 min. The second group were placed in the center of the OF (50 × 50 cm, 50 cm-high wall; Fig. 1C) to freely navigate through the arena for 10 min. The central 30 × 30 cm area of OF was selected as the more anxiogenic compartment, as previously recommended^43^. The animals’ activities in all behavioral conditions were recorded with a video camera, synchronized with electrophysiological recording. Of note, two rats in the EPM group were excluded from the experiment due to head-socket displacement. To quantify anxiety behaviors, the relative unsafe (or safe) exploration time as

$${\text{Relative unsafe }}\left( {\text{or safe}} \right){\text{ exploration}} = \frac{{{\text{Unsafe }}\left( {\text{or safe}} \right){\text{ exploration time}}}}{{\text{Total exploration time}}},$$

the relative unsafe (or safe) entries as

$${\text{Relative unsafe }}\left( {\text{or safe}} \right){\text{ entries}} = \frac{{{\text{Unsafe }}\left( {\text{or safe}} \right){\text{ entries}}}}{{\text{Total number of entries}}},$$

and the anxiety index as

$${\text{Anxiety Index}} = 1 - \frac{{\frac{{\text{Unsafe exploration time}}}{{\text{Total exploration time}}} + { }\frac{{\text{Unsafe entries}}}{{\text{Total number of entries}}}}}{2}$$

were computed as anxiety-related metrics.

### Electrode sites verification

Electrode sites were verified histologically as the following. First, the animals were deeply anesthetized with urethane (1.2 g/Kg, intraperitoneal injection). Prior to brain extraction, the recording sites were lesioned electrically. After extraction, the brains were fixated using %4 paraformaldehyde for 48 h. Electrodes’ sites were confirmed with light microscopy of 200-µm-thick coronal brain sections (Fig. 1A).

### LFP analysis

For the neurophysiological assessments, we analyzed each unsafe (open arms of EPM and central areas of OF) to safe (closed arms of EPM and peripheral areas of OF) transition. To do so, first we extracted the signal from 5 secs before to 5 secs after the transition. Next, each half of the signal was considered as one trial. The inverse transitions, that is safe-to-unsafe, were extracted and used similarly for Supplementary Fig. 6. For the familiar sessions, every 5-s period in which the animal had locomotion was used as a trial. To exclude the effect of locomotion on the regional neural activities, 5-s periods with movement were extracted, from EPM, OF, and baseline sessions, as previously suggested^11^. All subsequent analyses were performed on these mentioned trials.

PSD of LFPs were estimated by the Welch’s periodogram method (***pwelch*** function in MATLAB). This method applies Fourier transform on 1.76-s hamming windows sliding through the signal with 88% overlap; the average power corresponding to each frequency in each trial was considered as the raw power value. To highlight the frequencies with more prominent power changes, we computed relative power in theta range as the following. Raw power values of each frequency in every trial were divided by the sum of all power values from 0.1 to 200 Hz of the same trial. To exclude the between-subject differences in regional neural activities during EPM and OF sessions, the power values in each frequency is reported as its proportional increase compared to baseline session (Fig. 5). To do so, the average power value of each frequency during baseline trials was calculated for each rat separately. Next, every rat’s power values in the EPM and OF trials was divided by the corresponding rat’s baseline power, in a frequency- and trial-wise manner (baseline corrected power).

For the power correlation analysis, the spearman’s rank correlation was computed for the average of OB and mPFC relative power for each condition using MATLAB ***corr*** function. Magnitude-squared (MS) coherence between OB and mPFC signals was calculated using the MATLAB ***mscohere*** function. To exclude the effect of power enhancements on coherence results, we also reported PLV. To quantify PLV, we used the difference between mPFC and OB instantaneous phases, computed by wavelet transform, at every frequency and timepoint. This would create an (F × T) matrix of phase differences for every trial, where F is the vector of frequencies and T is the vector of timepoints. Next, the mean value of the phase difference matrix was stored for statistical comparisons. The cross-correlation between OB and mPFC theta range LFPs was calculated using the MATLAB ***xcorr*** function.

We used GC to measure the influx of information in the OB-to-mPFC and mPFC-to-OB directions. GC measures the predictability of one time-series based on another time-series using autoregressive models^38,39^. To do so, first, the data was down-sampled to 500 Hz, as recommended^44^. This effort improves the autoregressive models fitting and makes GC estimations more reliable^44^. Next, GC was calculated with the autoregressive models order 10. Such not-too-large model orders are more robust to noise^44^, which can lead to more accurate results. To statistically confirm the presence of GC in each of the conditions and directions, we computed the permuted distribution of GC in that case through shuffling time segments for 500 times. Next, the mean GC value from each condition was located on the distribution of the corresponding permutation results. To compare GC values between different conditions, mean GC value for every trial was calculated for further statistical analyses.

### Statistical analysis

Statistical analyses were implemented in MATLAB and GraphPad Prism (GraphPad Software, San Diego, CA, USA). Baseline and anxiogenic tests were compared by Mann–Whitney test. Wilcoxon signed rank test was used to compare relative exploration time of each compartment, relative entries to each compartment, GC between different directions as well as power, coherence, and cross-correlation between distinct states of each anxiogenic environment. We estimated the random distribution for GC by manual permutation in MATLAB. Computing the effect size (Cohen’s d) and Fisher r-to-z transformation was done using custom-written MATLAB functions. MATLAB ***corr*** function was used to compute the correlation between speed and theta oscillatory features, namely power and coherence. Fitting linear regression models for correlations was done using the MATLAB ***fitlm*** function. P-values less than 0.05 were considered as statistically significant.

### Supplementary Information

Supplementary Figure 1.

Supplementary Figure 2.

Supplementary Figure 3.

Supplementary Figure 4.

Supplementary Figure 5.

Supplementary Figure 6.

Supplementary Figure 7.

Supplementary Legends.

## Data Availability

Codes and Data will be made available upon reasonable request to the corresponding authors.
