# Supplementary figures and images for: Olfactory bulb-medial prefrontal cortex theta synchronization is associated with anxiety
Source: Sci Rep. 2024 May 27;14:12101. doi: 10.1038/s41598-024-63101-z (PMC11130310; doi:10.1038/s41598-024-63101-z)

Low Theta (4-8 Hz)

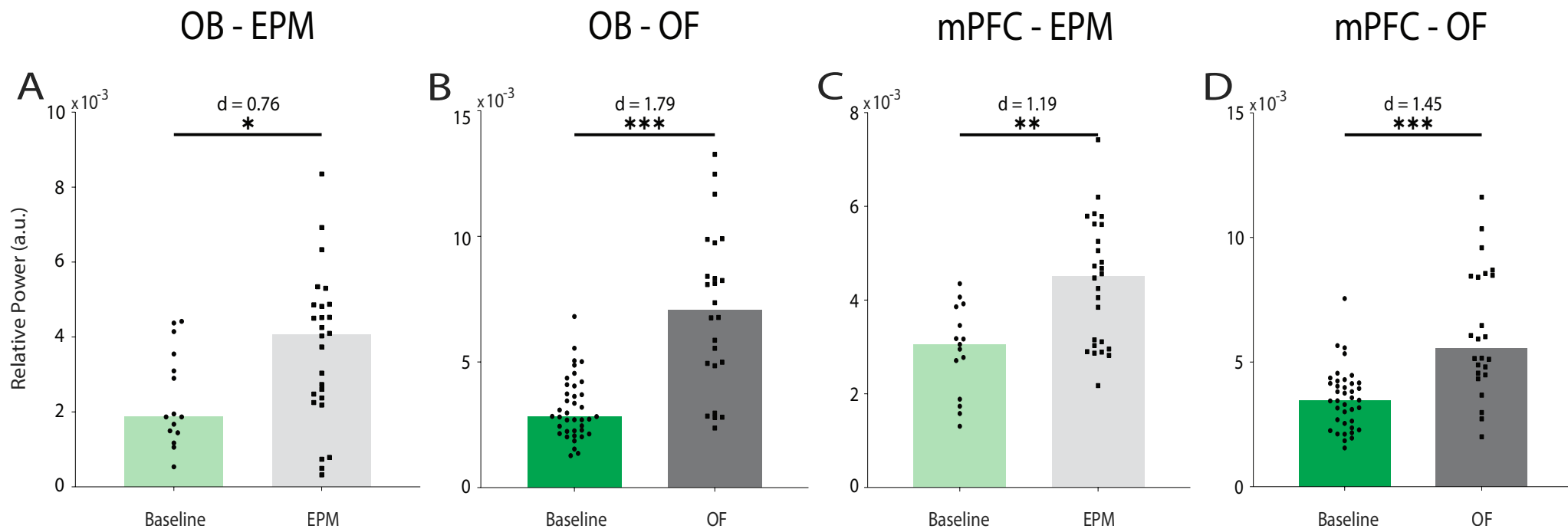

High Theta (8-12 Hz)

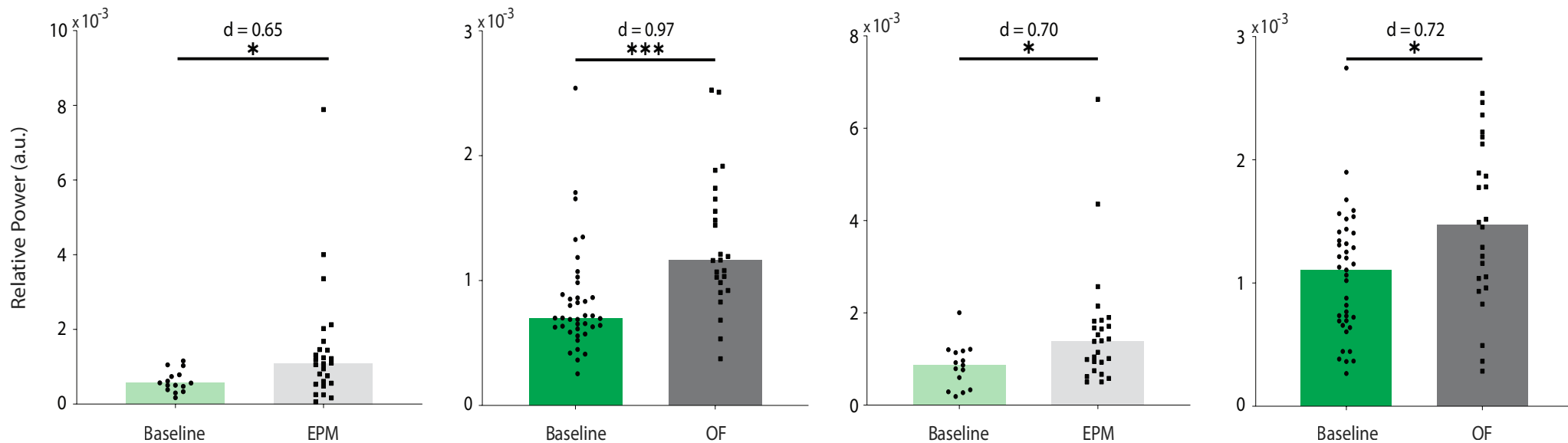

Supplement: Supplementary file 1 — Supplementary Figure 1. [file 41598_2024_63101_MOESM1_ESM.pdf]

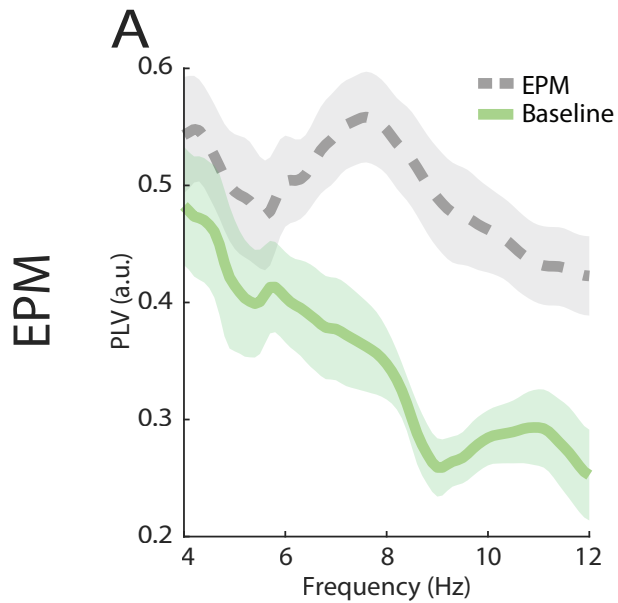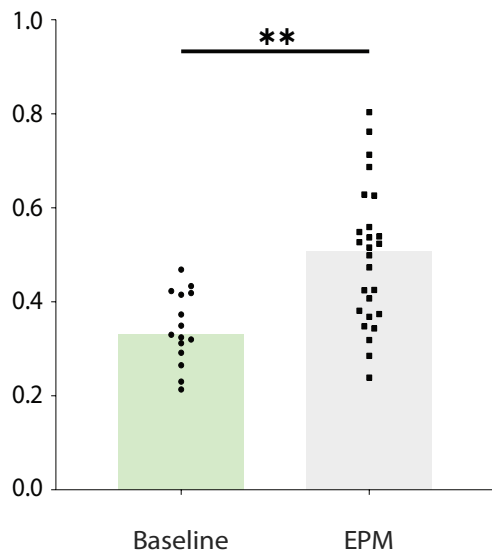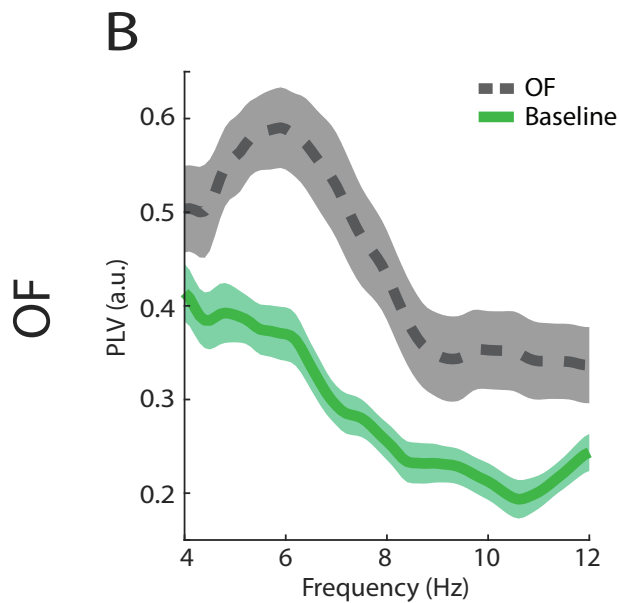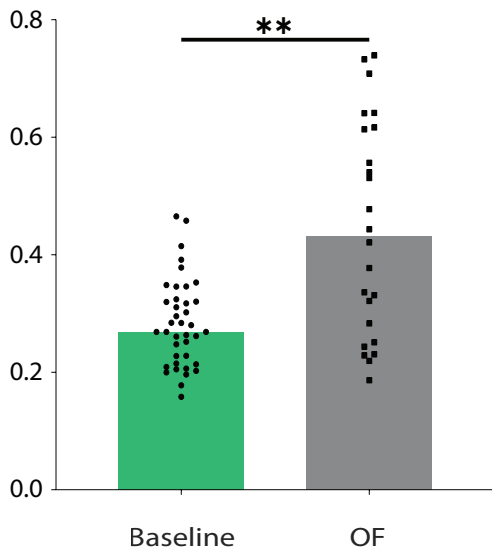

Supplement: Supplementary file 2 — Supplementary Figure 2. [file 41598_2024_63101_MOESM2_ESM.pdf]

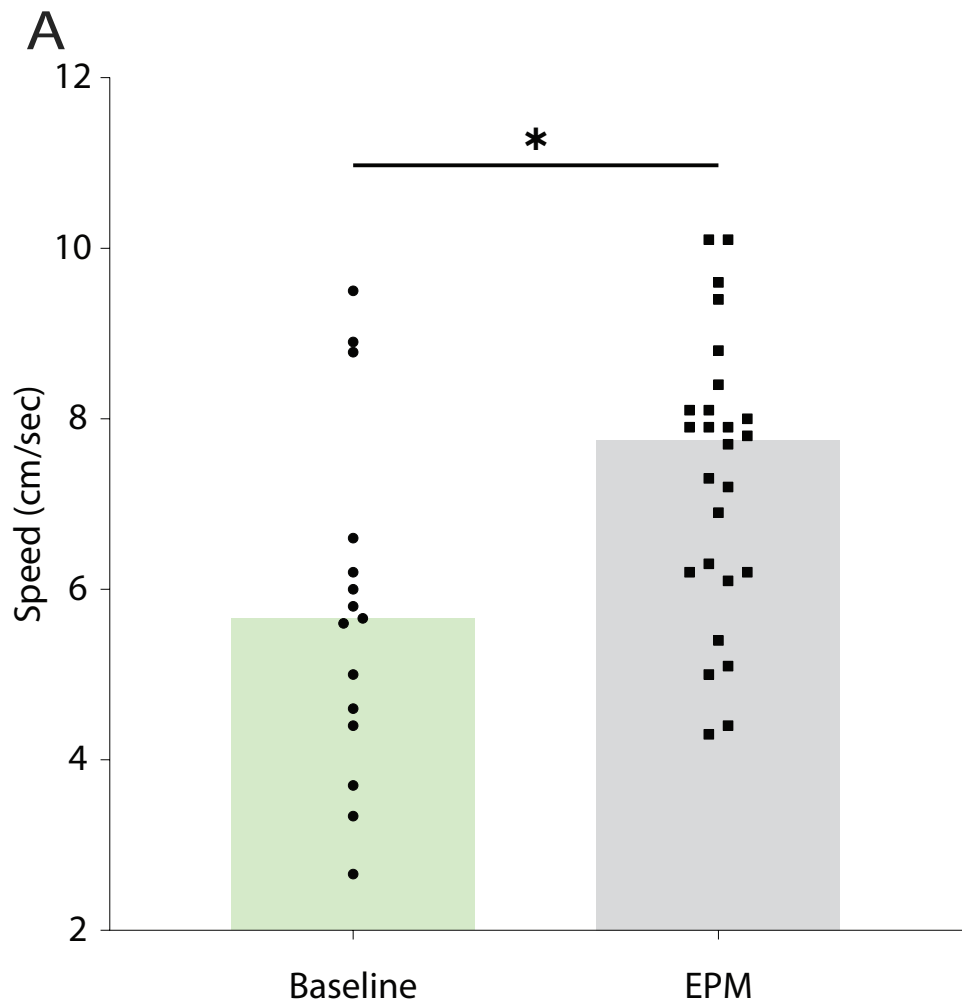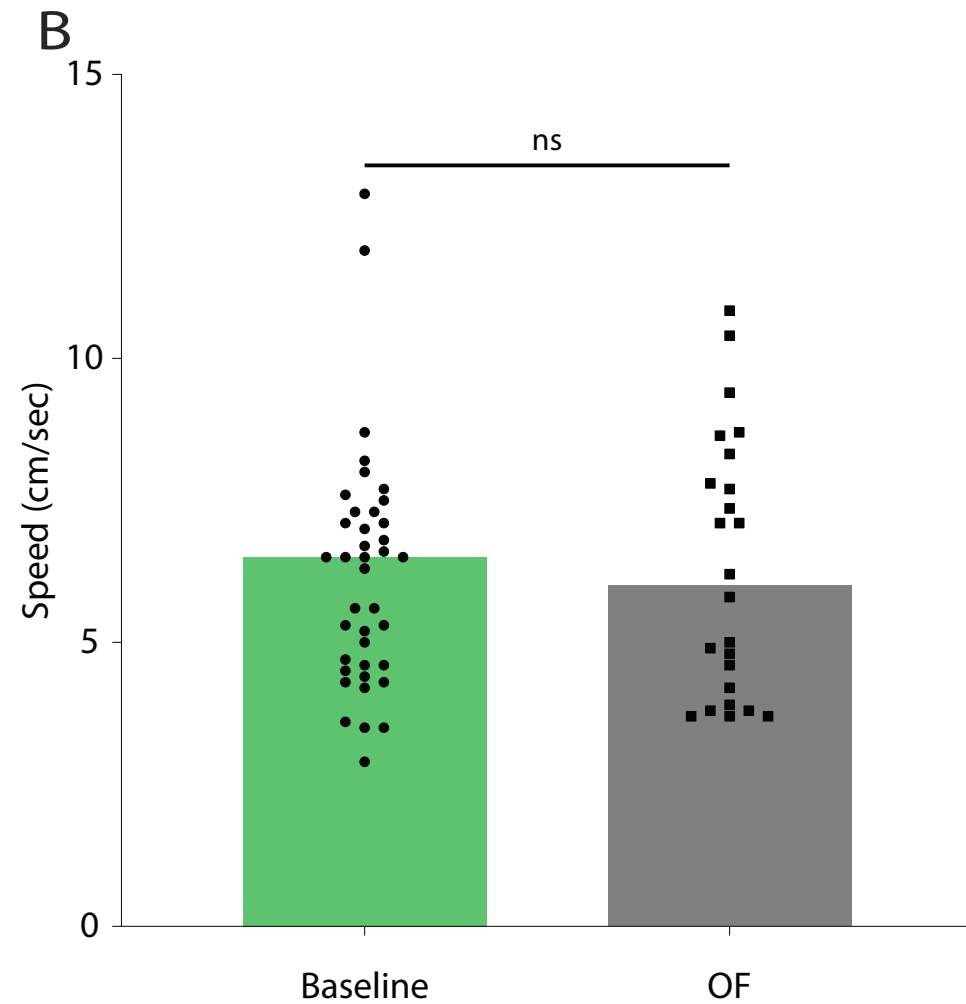

Supplement: Supplementary file 3 — Supplementary Figure 3. [file 41598_2024_63101_MOESM3_ESM.pdf]

Baseline

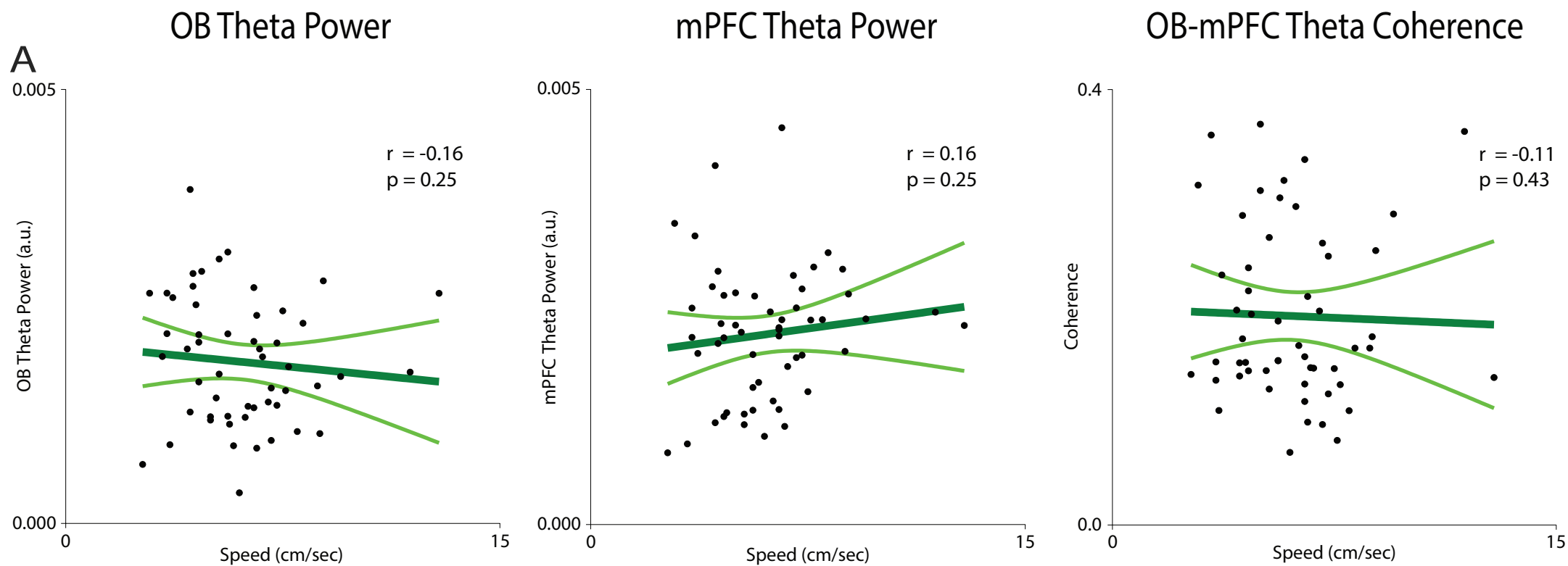

EPM

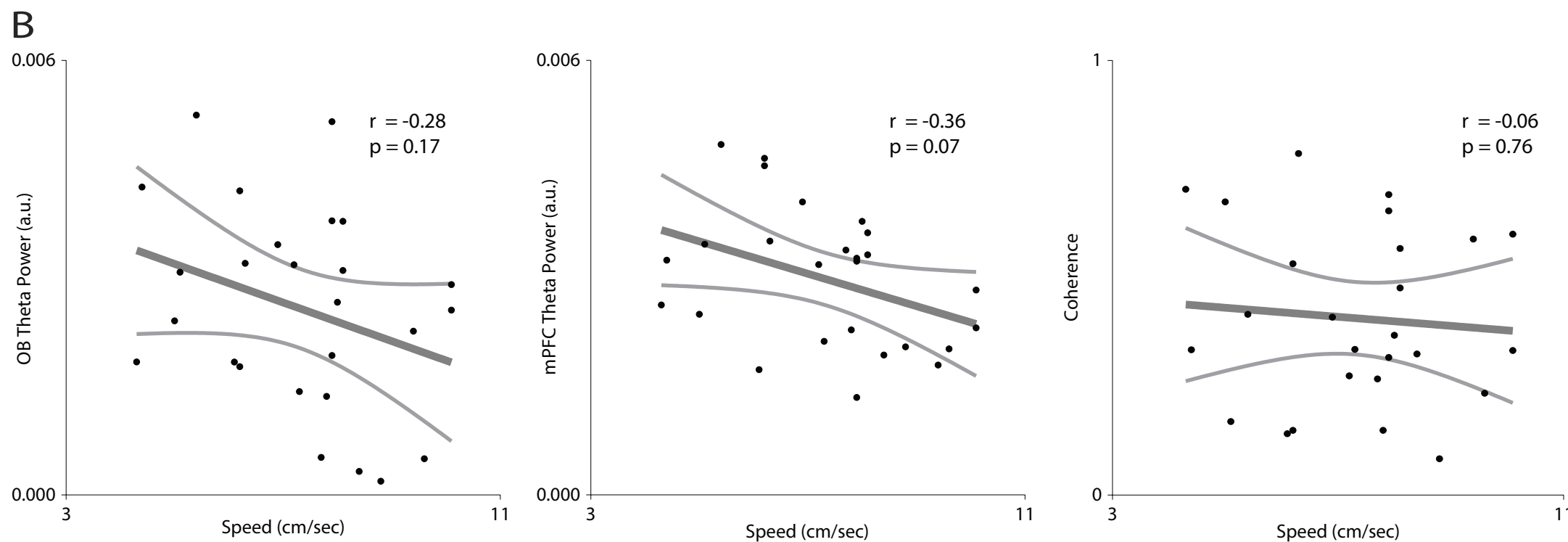

OF

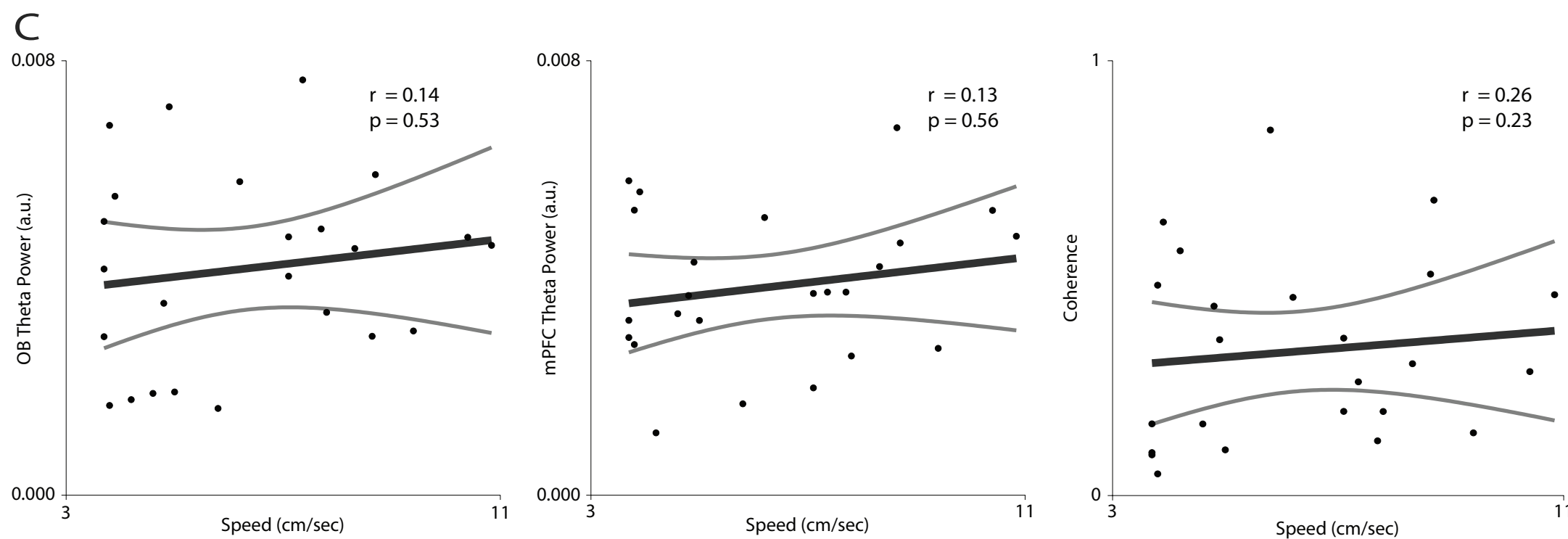

Supplement: Supplementary file 4 — Supplementary Figure 4. [file 41598_2024_63101_MOESM4_ESM.pdf]

OB  $\rightarrow$  mPFC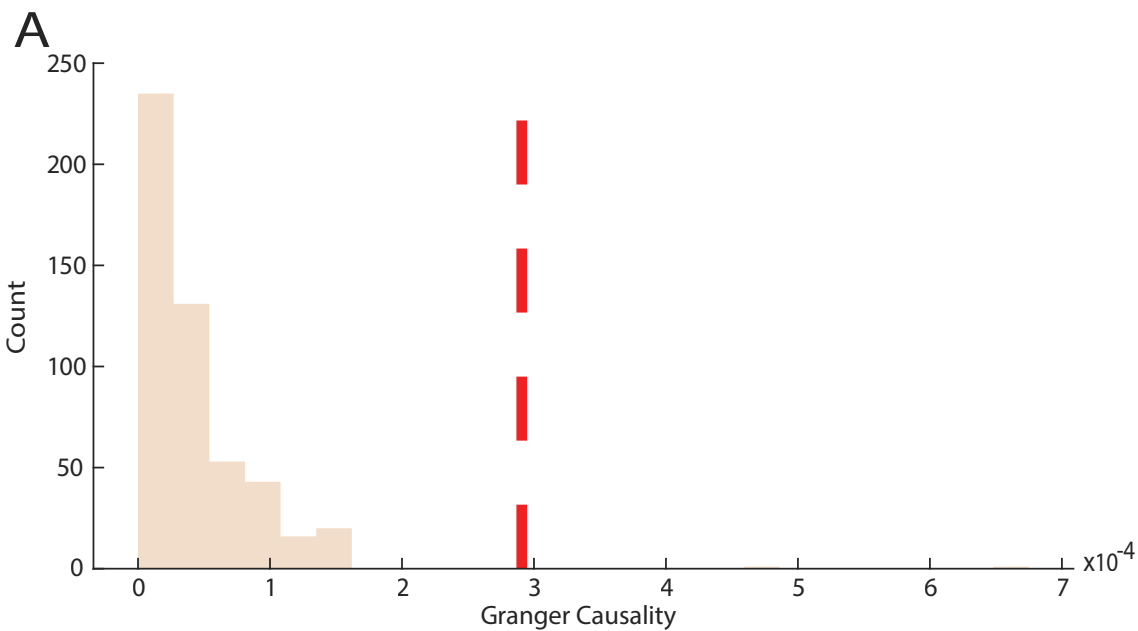mPFC  $\rightarrow$  OB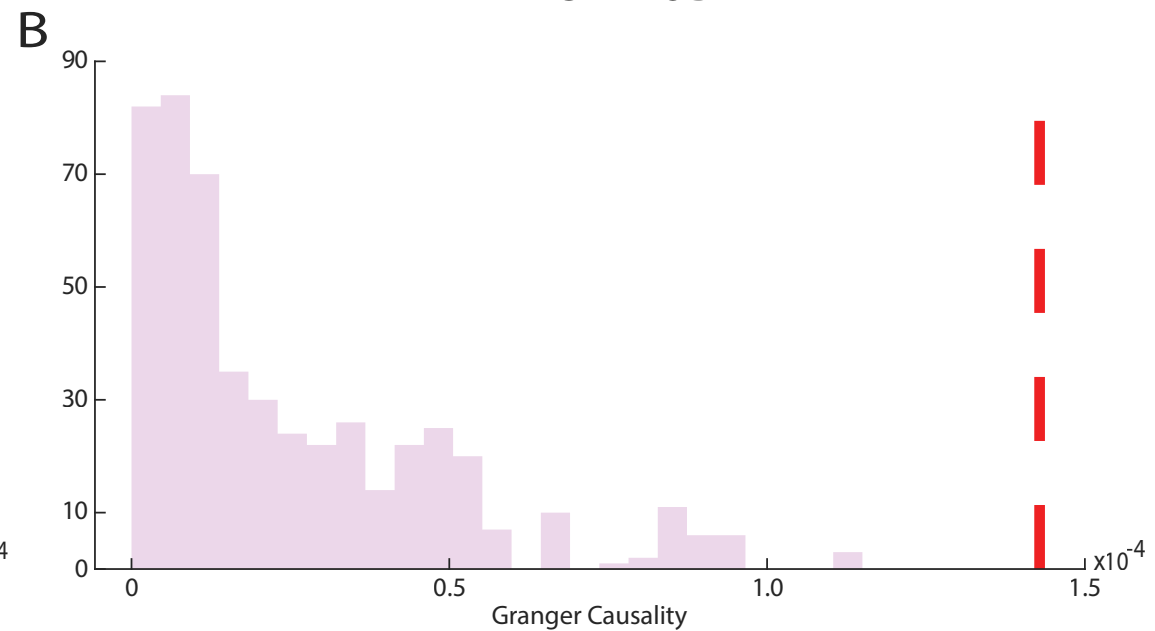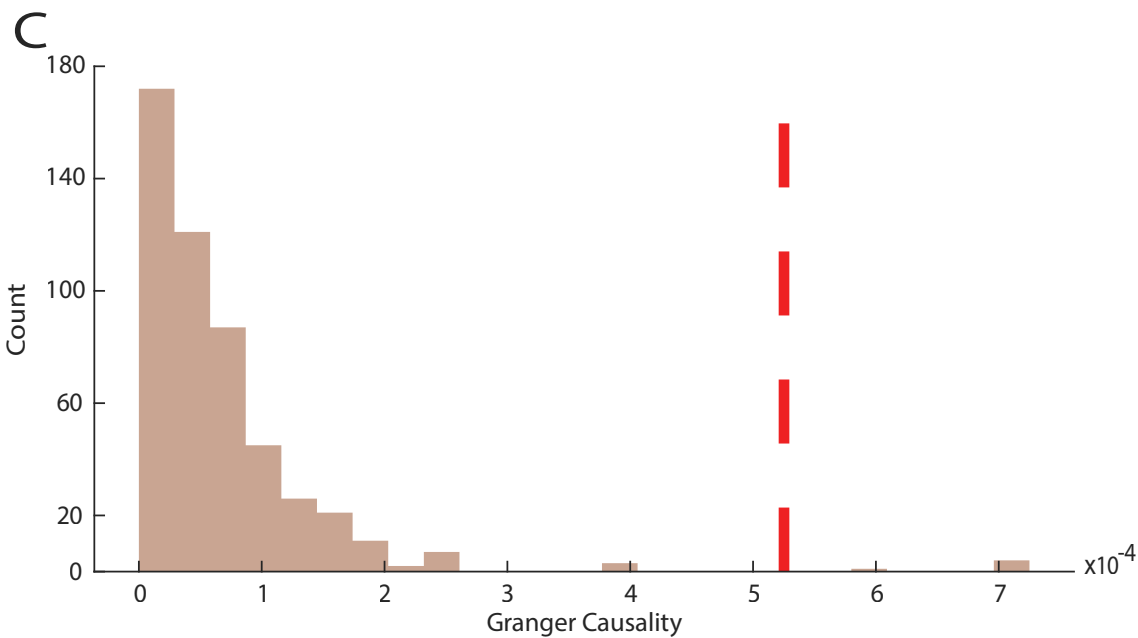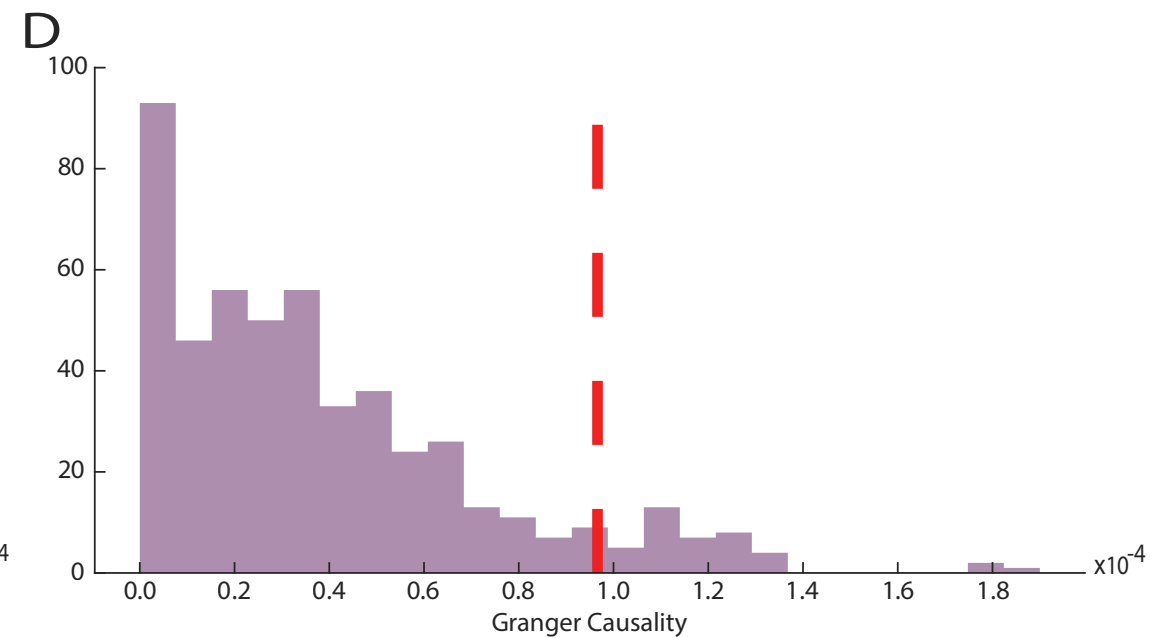

Supplement: Supplementary file 5 — Supplementary Figure 5. [file 41598_2024_63101_MOESM5_ESM.pdf]

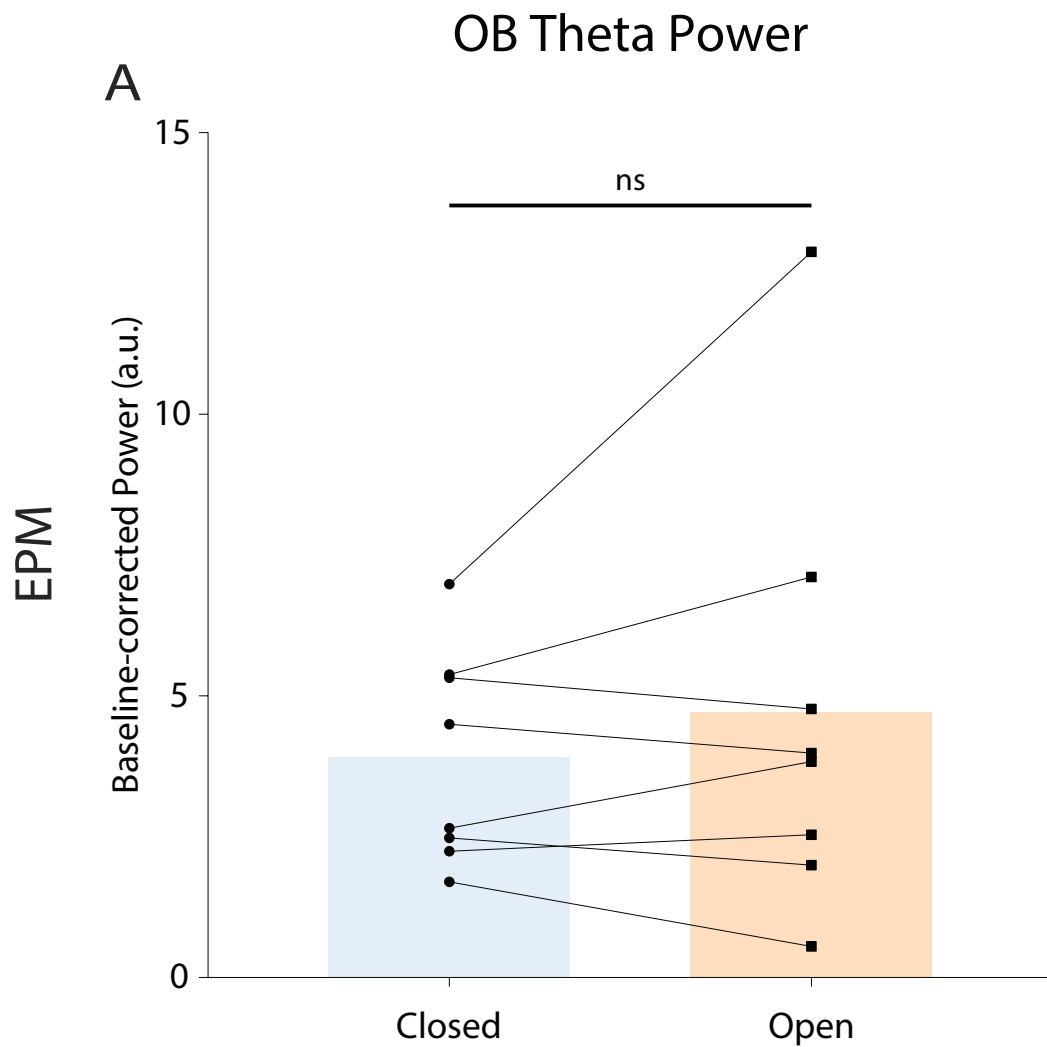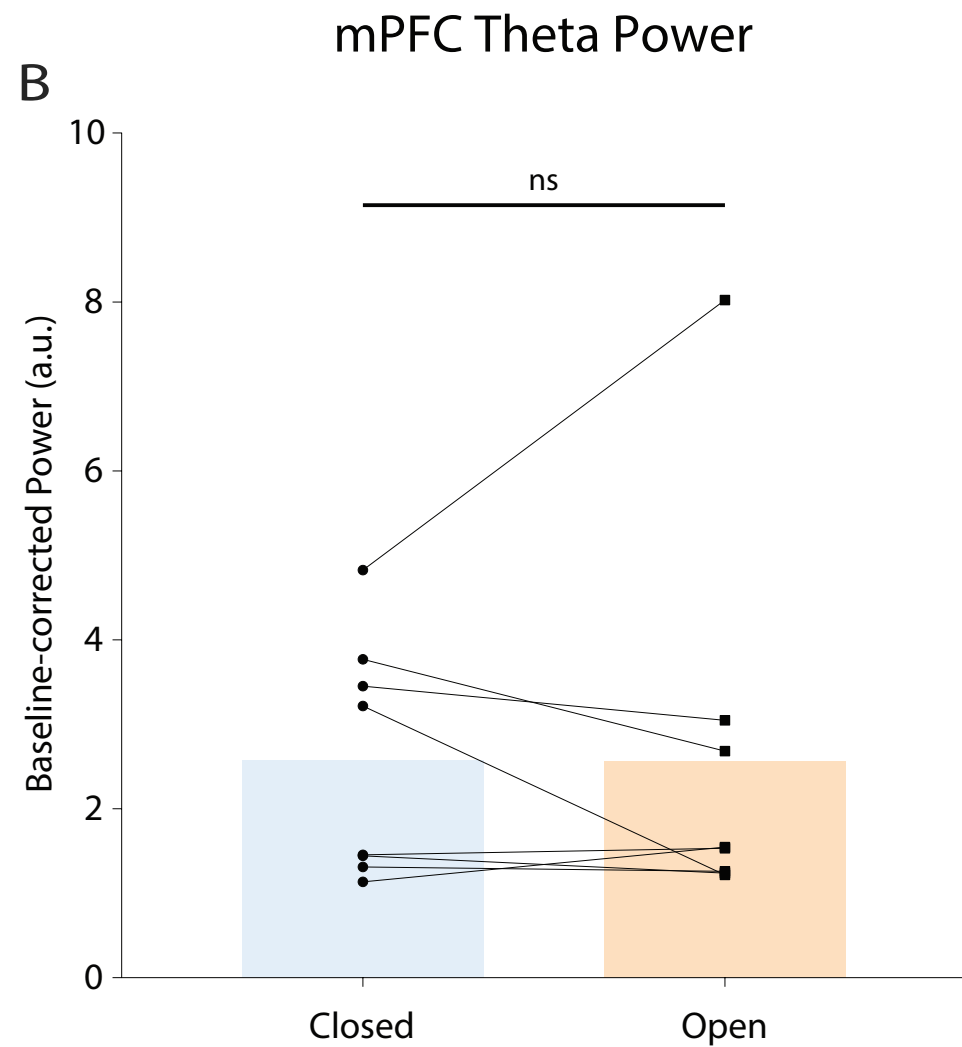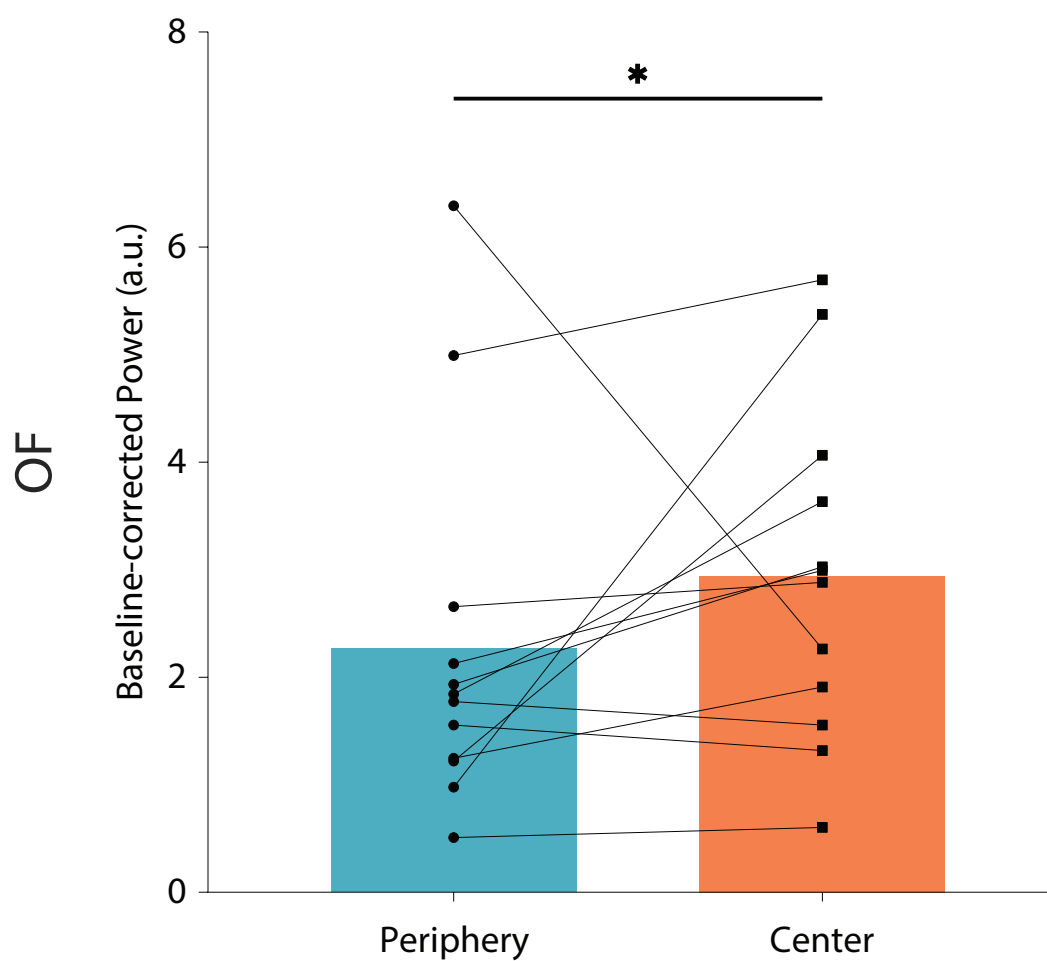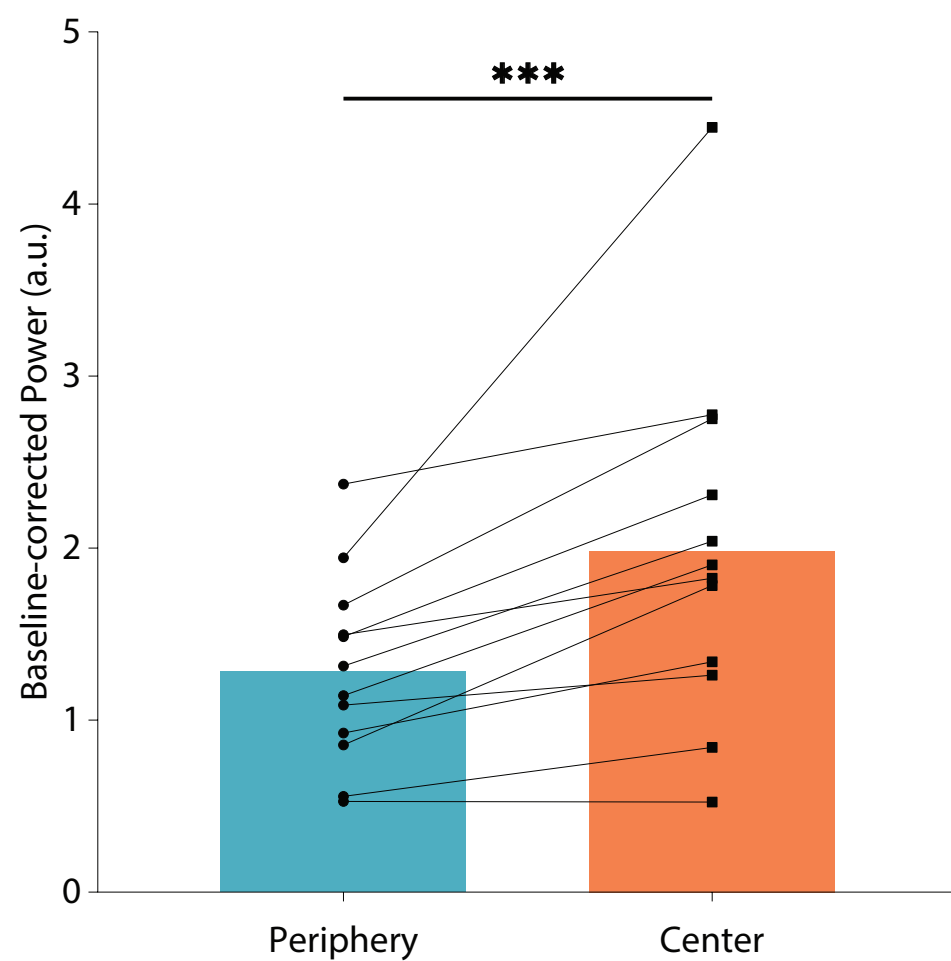

Supplement: Supplementary file 6 — Supplementary Figure 6. [file 41598_2024_63101_MOESM6_ESM.pdf]

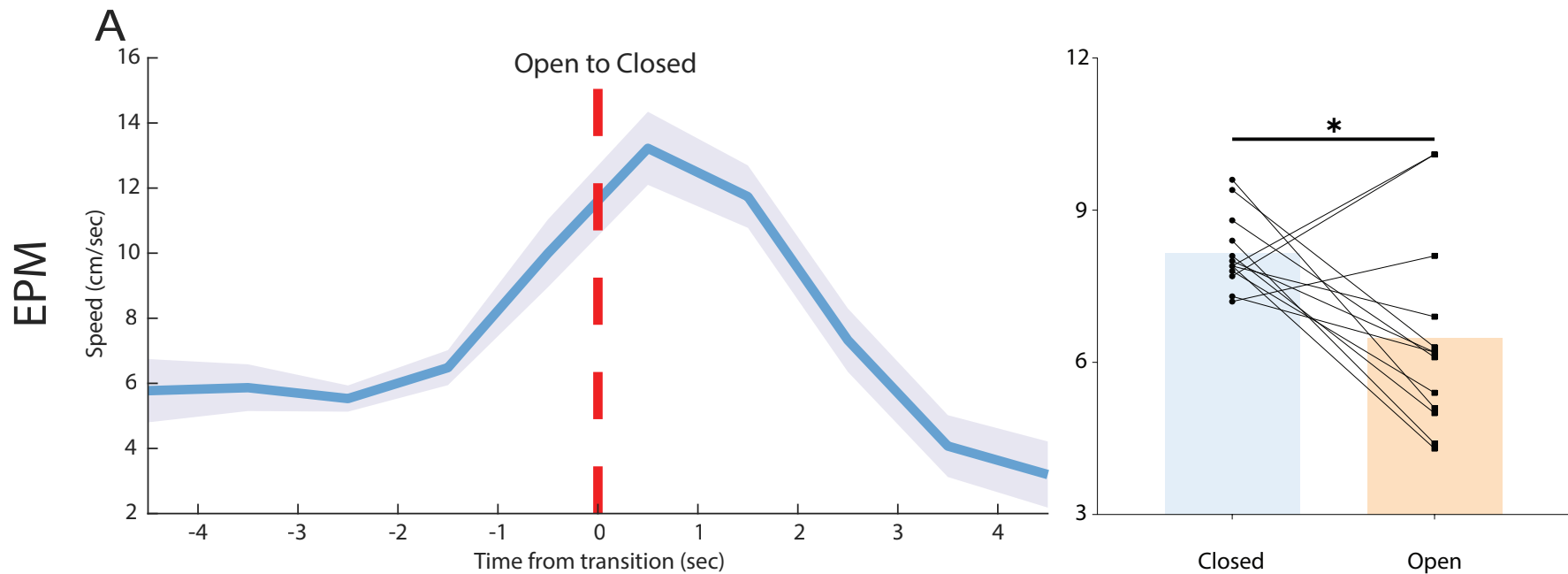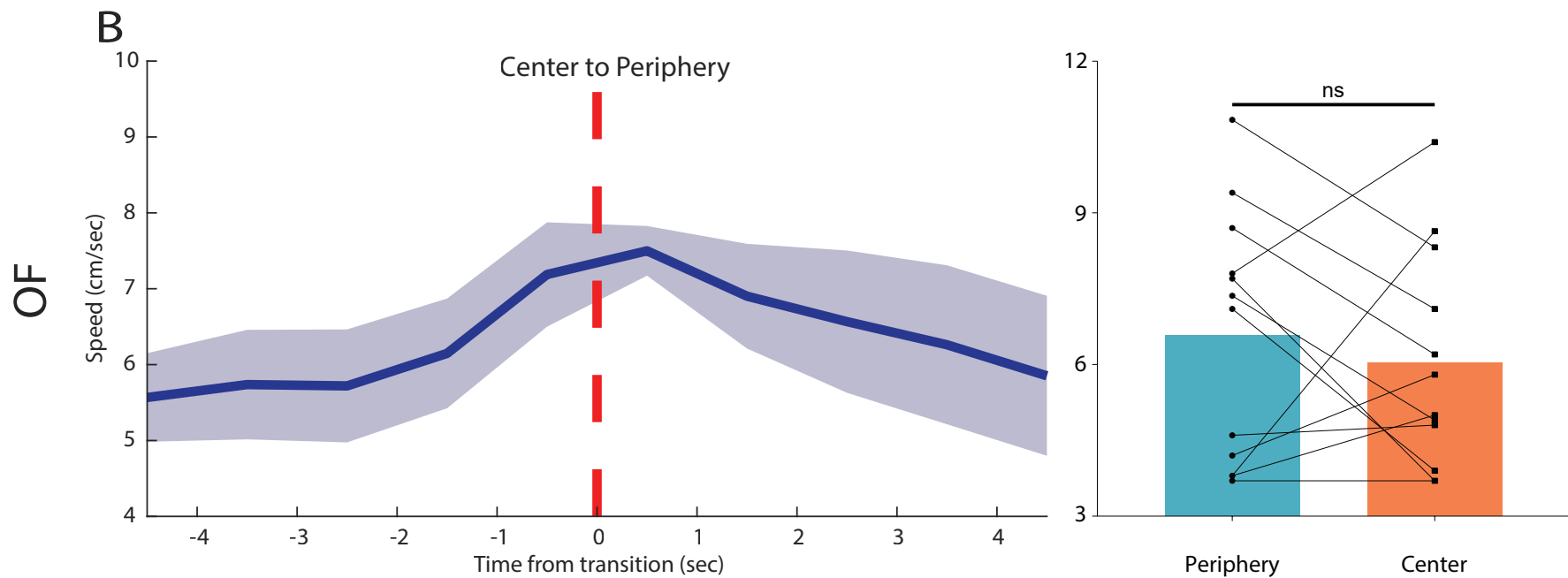

Supplement: Supplementary file 7 — Supplementary Figure 7. [file 41598_2024_63101_MOESM7_ESM.pdf]
