# Supplementary material for: Olfactory bulb-medial prefrontal cortex theta synchronization is associated with anxiety
Source: Sci Rep. 2024 May 27;14:12101. doi: 10.1038/s41598-024-63101-z (PMC11130310; doi:10.1038/s41598-024-63101-z)
Supplement: Supplementary file 8 — Supplementary Legends. [file 41598_2024_63101_MOESM8_ESM.pdf]

Supplementary Information

Olfactory bulb-medial prefrontal cortex theta synchronization is associated with anxiety

Morteza Mooziri, Ali Samii Moghaddam, Mohammad Ali Mirshekar*, Mohammad Reza Raoufy*

Supplementary Figure Legends

Supplementary Fig 1. Low vs. high theta power during anxiety. A-D Low Theta (upper panels) and high theta (lower panels) average power of OB (**A,B**) and mPFC (**C,D**) in the EPM (n = 26) (**A,C**) and OF (n = 24) (**B,D**) experiments (baseline: n = 15, n = 39, respectively). Bars represent median values in the bar plots. Statistical difference measured by Mann-Whitney test. *p < 0.05, **p < 0.01, ***p < 0.001. OB, olfactory bulb; mPFC, medial prefrontal cortex; EPM, elevated plus maze; OF, open field.

Supplementary Fig 2. OB-mPFC theta PLV during anxiety. A,B OB-mPFC theta spectral PLV (left panels) and average theta PLV (right panels) in EPM (n = 26) (**A**) and OF (n = 24) (**B**) (baseline: n = 15, n = 39, respectively). Data presented as mean ± SEM in the spectral PLV. Bars represent median values in the bar plots. Statistical difference measured by Mann-Whitney test. **p < 0.01. OB, olfactory bulb; mPFC, medial prefrontal cortex; PLV, phase-locking value; EPM, elevated plus maze; OF, open field.

Supplementary Fig 3. Animal's speed in the experiments. A,B Average animal's speed in EPM (n = 26) (**A**) and OF (n = 24) (**B**) experiments, (baseline: n = 15, n = 39, respectively). Bars represent median values. Statistical difference measured by Mann-Whitney test. *p < 0.05. EPM, elevated plus maze; OF, open field.

Supplementary Fig 4. Locomotion effects on OB-mPFC circuit theta oscillations. A-C Correlation between animal's speed and OB theta power (leftmost panels), mPFC theta power (middle panels), and OB-mPFC theta coherence (rightmost panels) during baseline (**A**), EPM (**B**) and OF (**C**). Each dot represents the average speed of the animal in a trial on the X axis versus the average theta power (leftmost and middle panels) or coherence (rightmost panels) of the same trial on the Y axis. Dark thick line indicates

fitted linear regression model. Light thin lines are %95 CI for the fitted linear regression model. OB, olfactory bulb; mPFC, medial prefrontal cortex; EPM, elevated plus maze; OF, open field. CI, confidence interval.

Supplementary Fig 5. GC permutation during anxiety. A-D Distribution of GC values for OB-to-mPFC (**A,C**) and mPFC-to-OB (**B,D**) in a random permutation procedure with 500 repetitions in EPM (**A,B**) and OF (**C,D**). Histograms show the distribution of permuted GC values in each case. Dashed line indicates the mean value obtained from data. GC, Granger causality; OB, olfactory bulb; mPFC, medial prefrontal cortex; EPM, elevated plus maze; OF, open field.

Supplementary Fig 6. OB and mPFC theta power during safe-to-unsafe transitions. A,B Average OB (**A**) and mPFC (**B**) theta power during trials of safe-to-unsafe transition in EPM (n = 8) (upper panels) and OF (n = 12) (lower panels) experiments. Bars represent mean values. Statistical difference measured by Wilcoxon signed rank test. *p < 0.05, ***p < 0.001. OB, olfactory bulb; mPFC, medial prefrontal cortex; EPM, elevated plus maze; OF, open field.

Supplementary Fig 7. Animal's speed in the anxiogenic environments. A,B Trace of animals' speed (left panels) and average speed (right panels) while exploring EPM (**A**) and OF (**B**). Data presented as mean ± SEM in the speed traces. Bars represent mean values in the bar plots. Statistical difference measured by Wilcoxon signed rank test. *p < 0.05. EPM, elevated plus maze; OF, open field.
